# SESN2 facilitates mitophagy by helping Parkin translocation through ULK1 mediated Beclin1 phosphorylation

**DOI:** 10.1038/s41598-017-19102-2

**Published:** 2018-01-12

**Authors:** Ashish Kumar, Chandrima Shaha

**Affiliations:** Cell Death and Differentiation Research Laboratory, National Institute of Immunology, New Delhi, India

## Abstract

Mitophagy, the selective degradation of mitochondria by autophagy, is crucial for the maintenance of healthy mitochondrial pool in cells. The critical event in mitophagy is the translocation of cytosolic Parkin, a ubiquitin ligase, to the surface of defective mitochondria. This study elucidates a novel role of SESN2/Sestrin2, a stress inducible protein, in mitochondrial translocation of PARK2/Parkin during mitophagy. The data demonstrates that SESN2 downregulation inhibits BECN1/Beclin1 and Parkin interaction, thereby preventing optimum mitochondrial accumulation of Parkin. SESN2 interacts with ULK1 (unc-51 like kinase 1) and assists ULK1 mediated phosphorylation of Beclin1 at serine-14 position required for binding with Parkin prior to mitochondrial translocation. The trigger for SESN2 activation and regulation of Parkin translocation is the generation of mitochondrial superoxide. Scavenging of mitochondrial superoxide lower the levels of SESN2, resulting in retardation of Parkin translocation. Importantly, we observe that SESN2 mediated cytosolic interaction of Parkin and Beclin1 is PINK1 independent but mitochondrial translocation of Parkin is PINK1 dependent. Together, these findings suggest the role of SESN2 as a positive regulator of Parkin mediated mitophagy.

## Introduction

As the main source of ATP, mitochondria is deemed to be a critical player in the regulation of cellular processes like death and survival^1,2^. The maintenance of the quality control through mitophagy has been highlighted as a protective cellular mechanism that controls the turnover of mitochondria^3,4^. Increasing evidences from past couple of decades have implicated mitophagy impairment in many diseases like cancer, metabolic diseases, inflammation, diabetes, neurodegradation and aging^5,6^. It is necessary to understand the molecular mechanisms of mitophagy in depth to develop therapeutic targets. Mitophagy is a selective autophagic process in which cytosolic Parkin which is a ubiquitin ligase, translocates and interacts with PINK1, a serine/threonine protein kinase located on the outer membrane of damaged mitochondria and thereby targets impaired mitochondria towards autolysosome for degradation^7^. For the clearance of damaged mitochondria, PINK1 has been shown to phosphorylate ubiquitin at serine-65 which enhances ligase activity and mitochondrial translocation of Parkin^8^. Deletion of PINK1 or Parkin results in mitochondrial dysfunction due to defective mitophagy indicating a central role of these molecular players in the functioning and turnover of mitochondria^9,10^. Recent reports have suggested that Beclin1 facilitates translocation of Parkin to damaged mitochondria during mitophagy, however mechanistic details of this process remains largely unknown^11^. However, it is unlikely that the entire process of mitophagy is restricted to these two molecules and there must be additional regulators that aid their functioning. Therefore, the regulation of these proteins in response to mitochondrial depolarisation or in the event of pathophysiological conditions, creates a complex scenario that needs to be investigated for a better understanding of the entire process.

Mitophagic clearance of aged/superfluous mitochondria is a stress dependent phenomenon, therefore, it is critical to address the role of stress induced regulatory proteins involved in this process. Sestrins are a highly conserved family of stress inducible antioxidant proteins present in 3 forms (SESN1, 2, 3) in mammals and are known to regulate autophagy and mitophagy related events in response to various cellular stresses^12–14^. Sestrins are homologous to bacterial peroxiredoxin reduction enzyme, AhpD and exhibit antioxidant functions with their expression regulated by p53^15^. SESN2 apart from its primary function (as an antioxidant) is involved in the regulation of AMPK-MTORC1 axis during genotoxic stress and was shown to regulate metabolic homeostasis^16,17^. In *Drosophila melanogaster*, Sestrin (dSESN) mediated regulation of AMPK-MTORC1 axis is critical to prevent chronic age related pathologies caused due to the repression of autophagic clearance of dysfunctional mitochondria by chronic activation of MTOR^18^. Current findings have defined upregulation of a mammalian Sestrin family member, SESN2 in response to ER stress and dysfunctional mitochondria^19–21^. A recent study has suggested the role of SESN2 mediated mitophagy in suppression of sepsis by inhibiting NLRP3 inflammasome activation^22^. Elevation in levels of SESN2 has been found in the mid brain of Parkinson’s patients (PD) while *in-vitro* neuroblastoma cells showed a protective role of SESN2 against 1-methyl-4-phenylpyridinium (MPP+) induced neurotoxicity^23^. However, the role of mammalian Sestrins in regulation of mitophagy and maintenance of quality control of mitochondria is not very well dissected.

Our findings shed light on a mechanistic role of SESN2 in the regulation of Parkin mediated mitophagy by aiding its translocation to the damaged mitochondria. The SESN2 regulates Parkin translocation by sensing an increase in CCCP-induced mitochondria generated superoxide and promotes mitophagy. In response to CCCP-induced mitochondrial damage, SESN2 facilitates Beclin1 and Parkin interaction through ULK1 mediated Beclin1 phosphorylation (serine-14) resulting in translocation of Parkin to the damaged mitochondria. Our data also show that PINK1 is essential for Parkin translocation, but is not necessary for the SESN2 dependent cytosolic interaction between Beclin1 and Parkin. The results suggest that during mitophagy, PINK1 primes the mitochondrial translocation of Parkin and acts as the very first impulse in the process, however it is SESN2 that facilitates this translocation by enhancing interaction between Parkin and Beclin1, which is independent of PINK1.

## Results

### SESN1 and SESN2 protect cells against CCCP induced mitochondrial damage

**Figure 1:**
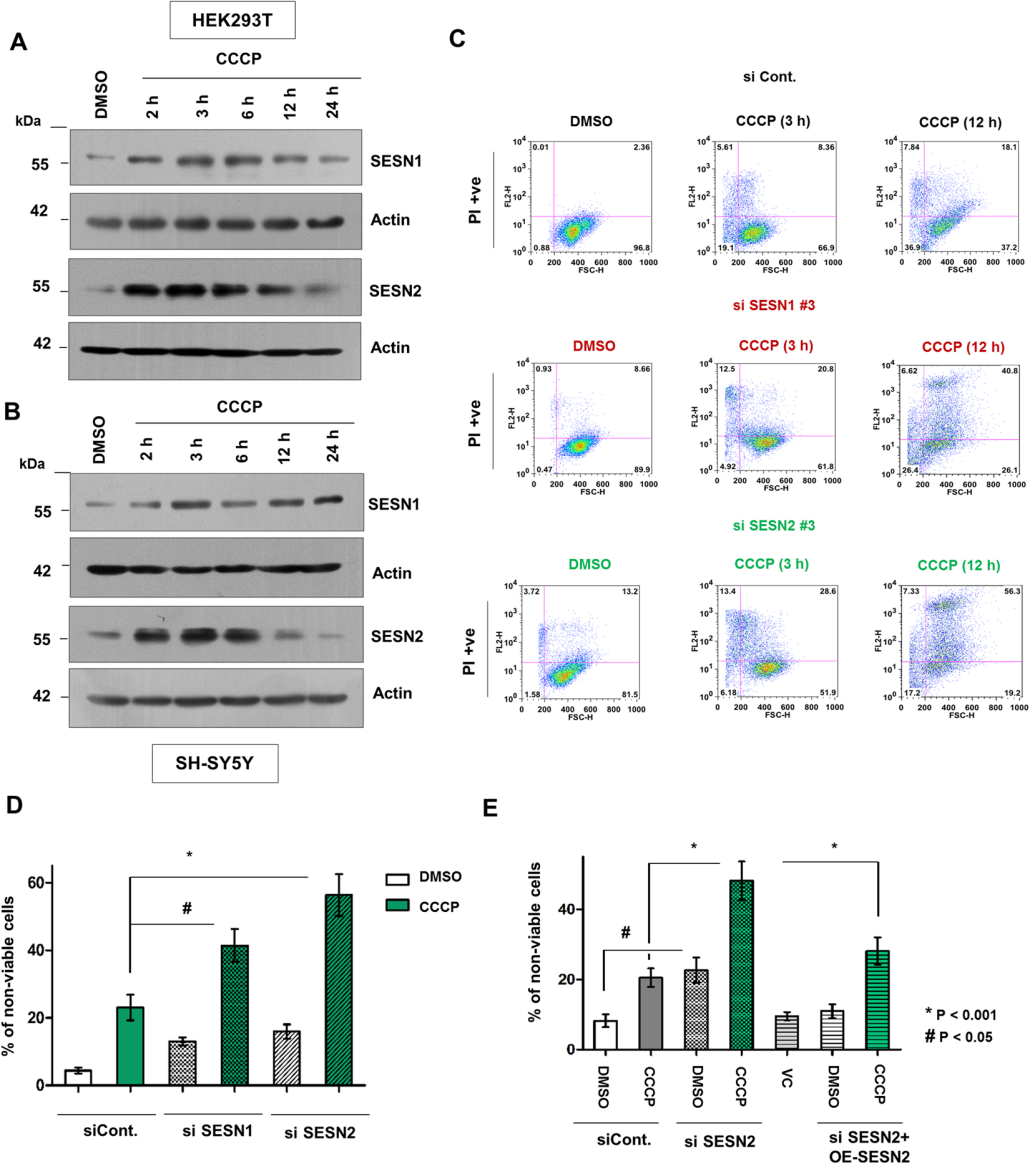
Sestrins protect cells from CCCP-induced mitochondrial damage. (**A**) HEK293T cells were treated with DMSO (vehicle control) and 10 µM CCCP for indicated time points and immunoblot analysis was done using indicated antibodies. Actin blot was used as a loading control. (**B**) SH-SY5Y cells were treated with DMSO (vehicle control) and 10 µM CCCP for indicated time points and immunoblot analysis was done using indicated antibodies. Actin blot was used as a loading control. (**C**) HEK293T cells were transfected with scrambled siRNA, siRNA targeting SESN1 and 2 followed by treatment with DMSO (vehicle control) and CCCP for 3 h and 12 h. Cell death analysis was done using PI assay. siSESN1 #3 and siSEN2 #3, si RNA to SESN1 and 2. (**D**) With similar experimental set up as performed in Fig. 1C, MTT assay was performed to measure the viability of cells. Error bars in the graph represent mean ± SEM from four independent experiments (n = 4). *p < 0.005, #p < 0.01. (**E**) Cell viability was measured using MTT assay, cells were transfected using scrambled siRNA, siRNA targeting SESN2 and plasmid overexpressing SESN2 as shown, followed by treatment with DMSO and CCCP (12 h). Error bars in the graph represent mean ± SEM from four independent experiments (n = 4). OE-SESN2, cells with overexpressed SESN2. *p < 0.001, #p < 0.05. Immunoblots were converted to gray and white scale using Adobe Photoshop CS6, with linear adjustment of exposure or contrast was done as per required. Images of immunoblots were cropped for clarity.

SESN1 and SESN2 are stress inducible proteins capable of functioning as antioxidants and participate in critical processes like autophagy and cellular metabolism^13,24^. Although their antioxidant property is well established, their role in mitochondrial homeostasis in not well defined. The purpose of this study was to establish the functional involvement of Sestrins, if any, in the maintenance of mitochondrial homeostasis. To address the questions, we primarily used HEK293T cells, a human embryonic kidney cell line and in some experiments SH-SY5Y cells derived from a neuroblastoma were used to confirm the results. For inducing mitochondrial stress, the cells were exposed to the protonophore CCCP which inflicts mitochondrial damage by uncoupling of the proton gradient^25^. In both cell lines, CCCP exposure induced a time dependent increase in the levels of both SESN1 and SESN2 till 6 h followed by a decline (Fig. 1A and B). The 10 µM CCCP dose was selected based on prior reports^7^ and the optimum degree of mitochondrial depolarization achieved after 3 h of exposure in HEK293T cells (Fig. S1A). The increase in SESN1 and SESN2 level is possibly due to an increase in expression rather than enhanced degradation as suggested by experiments using cycloheximide and a proteasomal inhibitor, MG132 (Kumar A. and Shaha C., 2017, Unpublished observation). The increase in expression of both SESN1 and SESN2 suggested possible functional involvement of the Sestrins during mitochondrial damage. To determine the relative importance of SESN1 and SESN2 proteins in the regulation of cellular function, both proteins were downregulated and cell death was assessed. Downregulation of both proteins resulted in increased cell death upon exposure to CCCP where SESN2 downregulation was comparatively more effective (Figs 1C,D and S2B). The importance of SESN2 was further evident from the experiment where knockdown of SESN2 markedly increased CCCP mediated cell death while overexpression of the SESN2 rescued cells from death (Fig. 1E). To explore if other mitochondrial damaging agents like oligomycin and antimycin A showed similar effects, cells were treated with the two agents that resulted in mitochondrial damage and influenced SESN2 activation in a similar way as with CCCP (Fig. S2C). In summary, this section demonstrates that Sestrins are involved in cellular protection against CCCP induced mitochondrial depolarization in both a kidney and a neuroblastoma cell line. SESN2 selected for further investigation also shows its protective action when oligomycin/antimycin combination was given.

### SESN2 mediates mitochondrial translocation of Parkin to damaged mitochondria

**Figure 2:**
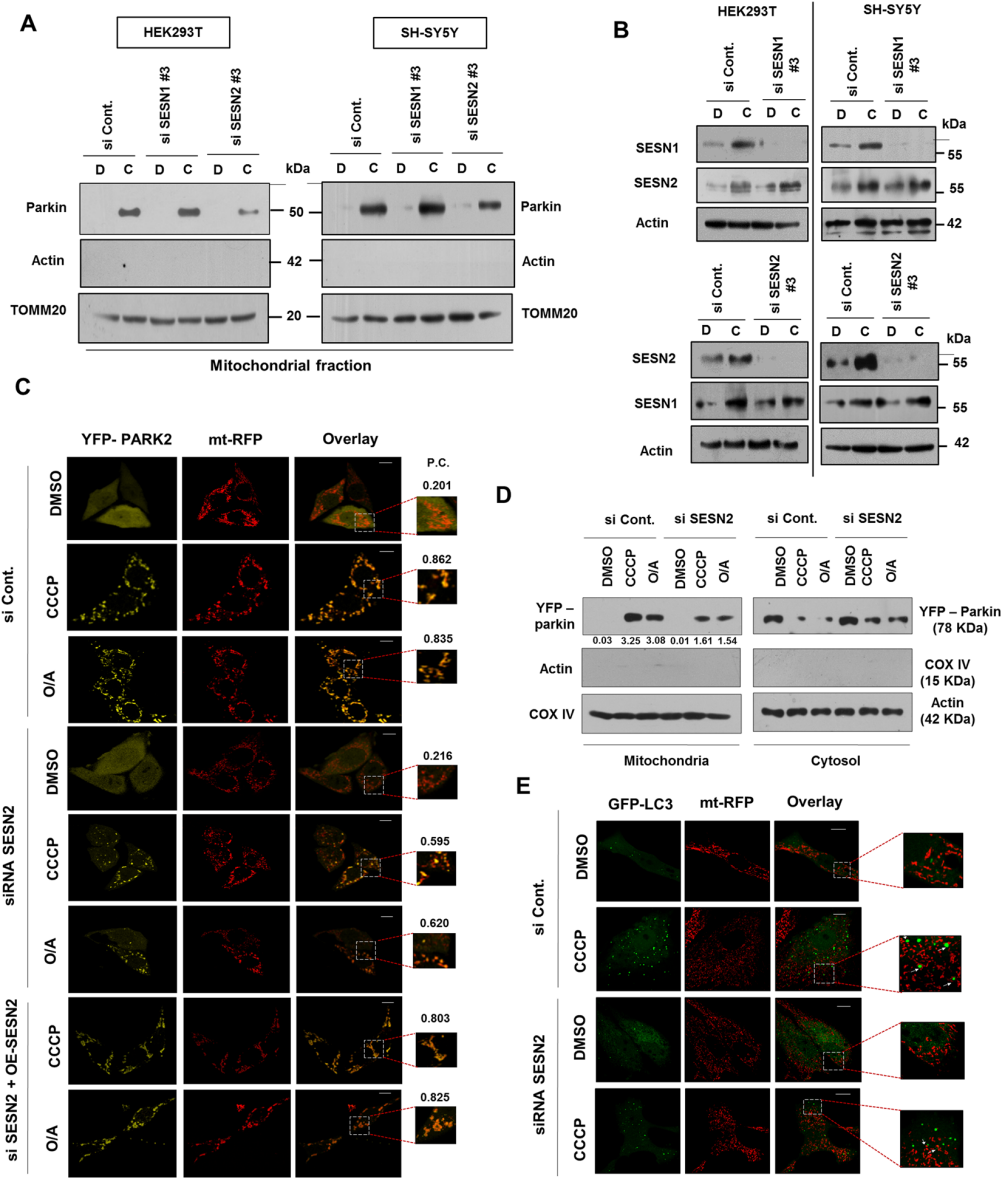
SESN2 is required for translocation of Parkin to the damaged mitochondria. (**A**) HEK293T and SH-SY5Y cells were transfected with scrambled siRNA and siRNA targeting SESN1 and 2 (siRNA #3) followed by exposure with 10 µM CCCP for 3 h. Post-treatment, isolated mitochondrial fractions were analysed to check translocation of Parkin to the mitochondria. D, DMSO and C, CCCP. (**B**) Whole cell lysates were measured for the levels of SESN1 and 2. TOMM 20 was used as mitochondrial marker while Actin was used as cytosolic marker. D, DMSO and C, CCCP (3 h). (**C**) HeLa cells stably expressing YFP-Parkin were transfected using mitochondria matrix-targeted RFP plasmid, scrambled siRNA and siRNA targeting SESN2. Treatment with 10 µM CCCP for 3 h and 5 µM Oligomycin + 5 µM antimycin A for 2 h was given and co-localization was scored. Pearson’s co-localization coefficient, P.C. > 0.7, significant. Scale Bars, 10 µm. (**D**) YFP-Parkin levels were analysed in mitochondria and cytosolic fractions of wild-type and SESN2 knockdown HeLa cells (YFP-Parkin expressing) which were treated with 10 µM CCCP for 3 h and 5 µM Oligomycin + 5 µM antimycin A for 2 h. Relative density (Mitochondria/Cytosol) of YFP-parkin as per represented in figure as numerical values. COX IV, mitochondrial marker; Actin, cytosolic marker. (**E**) Cells were sorted for mt-RFP signal (as mentioned earlier) and LC3-GFP plasmid was transfected. Confocal microscopy was done to check accumulation of LC3 puncta on mitochondria in wildtype and SESN2 knockdown cells. Immunoblots were converted to gray and white scale using Adobe Photoshop CS6, with linear adjustment of exposure or contrast was done as per required. Images of immunoblots were cropped for clarity, expanded view of Fig. 2A is available in supplementary figures.

Having established that reduction of Sestrins increased cell death on disruption of mitochondrial potential (∆Ψ_m_), we sought to understand if these proteins played any role in CCCP-induced mitophagy. The pivotal event for mitophagy is the translocation of the cytosolic Parkin to the mitochondria. To determine if sestrins had a role in this, both SESN1 and 2 were downregulated during CCCP treatment and mitochondrial fractions were checked. The analysis of isolated mitochondrial fractions showed a significant decline in the mitochondrial translocation of Parkin in SESN2 knockdown cells at 3 h of CCCP treatment (Fig. 2A). Suppression of SESN1 expression did not affect the translocation of Parkin suggesting involvement of SESN2 but not SESN1 in the process of mitochondrial translocation of Parkin (Fig. 2A). To be sure that SESN1 level was not affected by SESN2 downregulation and vice versa, levels of both proteins were checked in whole cell lysates of both cell types. SESN1 downregulation did not affect SESN2 and vice versa (Fig. 2B). Keeping in view the death of higher percentage of cells and inhibition of Parkin translocation when SESN2 was downregulated, the functional role of SESN2 was selected for further investigations. To further check the role of SESN2 in localization of Parkin, HeLa cells stably expressing YFP-tagged Parkin were treated with CCCP and oligomycin + antimycin A (O/A). HeLa cells were used as they lack endogenous Parkin and live cell imaging using mitochondrial matrix-associated mt-RFP plasmid as mitochondrial marker was done. We found that Parkin failed to translocate efficiently to the damaged mitochondria on downregulation of SESN2 expression upon both CCCP and oligomycin + antimycin A (O/A) treatment (Fig. 2C) while overexpression of the SESN2 restored the accumulation (Figs 2C and S2C), confirming the role SESN2 in mitochondrial translocation of Parkin. Moreover, mitochondrial fractionation post-treatment with oligomycin and antimycin A validated the involvement of SESN2 in mitochondrial translocation of Parkin (Fig. 2D). Cell imaging using TOMM20 (outer mitochondrial membrane translocase 20) as mitochondrial marker has confirmed the above results with inhibition of mitochondrial accumulation of Parkin on SESN2 downregulation upon CCCP treatment which was rescued on SESN2 overexpression (Fig. S2A-C and F). Arguably, the lack of Parkin accumulation to the mitochondria could be due to decrease in total protein levels. However, data showed that the translocation of Parkin on SESN2 knockdown was not due to decrease in protein level of Parkin (Fig. S2D). Consistent with observations from other reports, CCCP exposure for longer time (24 h) showed a decline in Parkin translocation to the mitochondria (Fig. S2E and F). To check if SESN2 mediated decline in Parkin translocation also affects CCCP or O/A induced mitophagy, we checked accumulation of LC3 puncta with mitochondria in wildtype and SESN2 downregulated cells. A substantial decline in LC3 puncta formation and its accumulation with mitochondria was observed in SESN2 downregulated cells on CCCP treatment (Figs 2E and S2G). To confirm our results, we did mito-keima assay as per performed previously^26^. Our results showed that SESN2 downregulation declines lysosomal targeting of mitochondria after CCCP or O/A treatment (12 h) which defines that SESN2 downregulation also supresses mitophagic process (Fig. S2H). Since, SESN2 downregulation resulted an increase in cell death, we investigated if inhibition in Parkin translocation was due to increase in the number of apoptotic cells. To decipher the same, we sorted out annexin positive and annexin negative population (Fig. S2I) and analysed mitochondrial fractions for YFP-Parkin translocation (Fig. S2J). Our results showed that both apoptotic and non-apoptotic cells have lesser Parkin translocation to mitochondria as compared to wild type, suggesting that inhibition in Parkin translocation was independent of the status of the cell. Taken together, these results clearly suggested participation of SESN2 in regulating the migration of Parkin to the damaged mitochondria.

### CCCP mediated mitochondria generated superoxide acts as a signal for SESN2 activation

**Figure 3:**
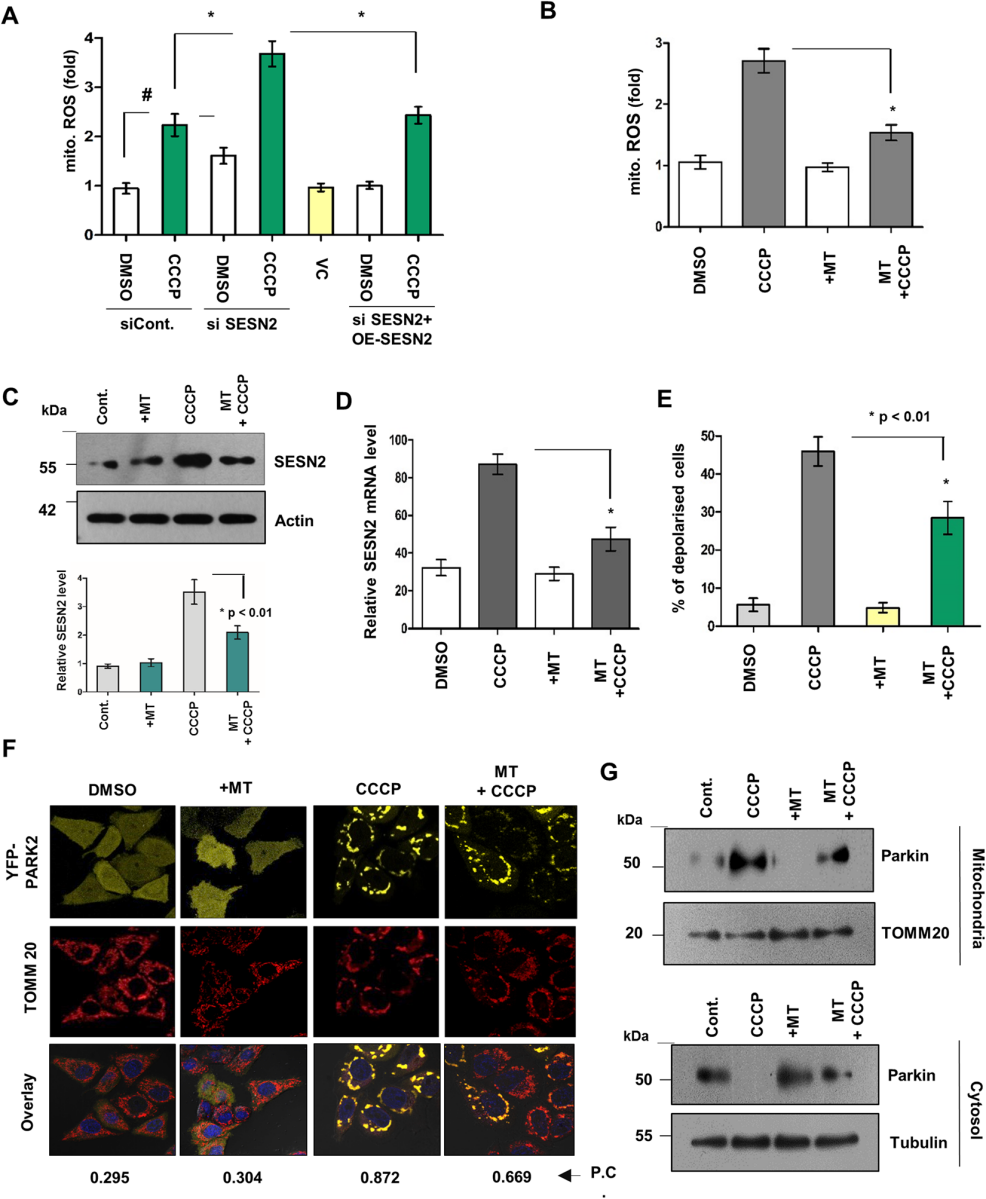
Increase in mitochondrial ROS is a signal which activates SESN2 and mitochondrial translocation of Parkin. (**A**) HEK293T cells were transfected with scrambled siRNA (siCont.), siRNA SESN2 (siSESN2) and SESN2 overexpression plasmid (OE-SESN2) followed by treatment with CCCP for 3 h. Mitochondrial ROS was detected by mitoSOX assay and compared with controls. Detection and quantification of mitochondrial ROS was done by flourometry and represented as fold change in intensity. Error bars in the graph represent mean ± SEM from four independent experiments (n = 4). *p < 0.01, #p < 0.05. (**B**) HEK293T cells were treated with mitochondrial superoxide quencher, mito-Tempo (MT, 500 µM) for 30 min. prior to 2 h CCCP (10 µM) treatment and measurement in fold decline of mitochondria generated ROS was carried out by performing mitoSOX assay and detected using flourometry. Error bars, SEM from four independent experiments (n = 4). *p < 0.01 C. Using same experimental setup as above, western blot analysis of SESN2 was done. Results show substantial reduction in CCCP-induced SESN2 protein levels on application of mito-Tempo. Densitometry of protein bands obtained in Fig. 3C and SESN2 to Actin ratio represents as relative SESN2 intensity. Error bars, mean ± SEM of three independent experiments (n = 4). *p < 0.01. (**D**) Measurement of mRNA levels of SESN2 in similar experimental setup as of Fig. 3C was done by reverse transcriptase PCR (RT-PCR). Error bars in the graph represent mean ± SEM from three independent experiments (n = 3). *p < 0.01. (**E**) HEK293T cells were treated with mito-Tempo and CCCP as indicated above. JC-1 staining and detection of green to red signal ratio (535/595 nm) was scored by fluorimeter. Error bars represent the mean ± SEM (n = 3). *p < 0.01 F. HeLa cells stably expressing YFP-Parkin were treated with CCCP and mito-Tempo and co-localization of YFP-Parkin with mitochondrial marker TOMM20 was observed. Note: CCCP induced mitochondrial translocation of Parkin significantly reduced on MT treatment. Scale Bars, 10 µm. Pearson’s co-efficient (P.C.), ≥ 0.7, significant. (**G**) To validate the results obtained, cellular fractionation was done with HEK293T cells similar experimental settings as above. Mitochondrial and cytosolic fractions were analysed for mitochondrial accumulation of Parkin by immunoblotting. (**H**) Western blot analysis of total amount of YFP-Parkin was scored in HeLa cells stably expressing Parkin as indicated. Immunoblots were converted to gray and white scale using Adobe Photoshop CS6, with linear adjustment of exposure or contrast was done as per required. Images of immunoblots were cropped for clarity, expanded view of Fig. 3C is available in supplementary information.

Next, to determine the possible signals sensed by cytosolic SESN2 in order to promote the mitochondrial migration of Parkin, we sought to understand the role of mitochondria generated ROS in mitophagy. Treatment with CCCP increased the generation of mitochondrial superoxide, however, this increase was much higher in SESN2 downregulated cells whereas overexpression of SESN2 significantly reduced the levels (Fig. 3A), suggesting an association of SESN2 with intracellular levels of mitochondria generated ROS. To figure out whether mitochondria generated superoxide acts as a signal for SESN2 activation, cells were treated with mito-Tempo (MT), a quencher specific for mitochondria generated superoxide (Fig. 3B). Quenching of mitochondrial superoxide with MT also reduced the intracellular protein and mRNA levels of SESN2 upon CCCP treatment (Fig. 3C and D). We found that treatment of mito-Tempo decline the extent of CCCP-induced depolarisation of cells (Fig. 3E). To further confirm if mitochondria generated superoxide could assist the migration of Parkin, microscopic evaluation of translocation of YFP-tagged Parkin was carried out with cells preincubated with MT prior to CCCP exposure. Results showed that application of mito-Tempo markedly inhibits mitochondrial translocation of Parkin induced by CCCP (Figs 3F and S3A) or other mitochondrial damaging agents like antimycin A and paraquat (PQ) (Fig. S3B and C). Further, validation of above results carried out by comparing mitochondrial fractions for Parkin in CCCP treated and MT pre-treated cells showed a significant decline in the amount of Parkin in MT pre-treated cells as compared to CCCP treated cells (Fig. 3G). Analysis of total amount of YFP-Parkin shows that decline in mitochondrial translocation of Parkin is not due to reduction in its total amount (Fig. 3H). Collectively, these results suggest the importance of mitochondria generated superoxide in activation of SESN2.

### SESN2 promotes interaction between Beclin1 and Parkin to facilitates mitochondrial translocation of Parkin

**Figure 4:**
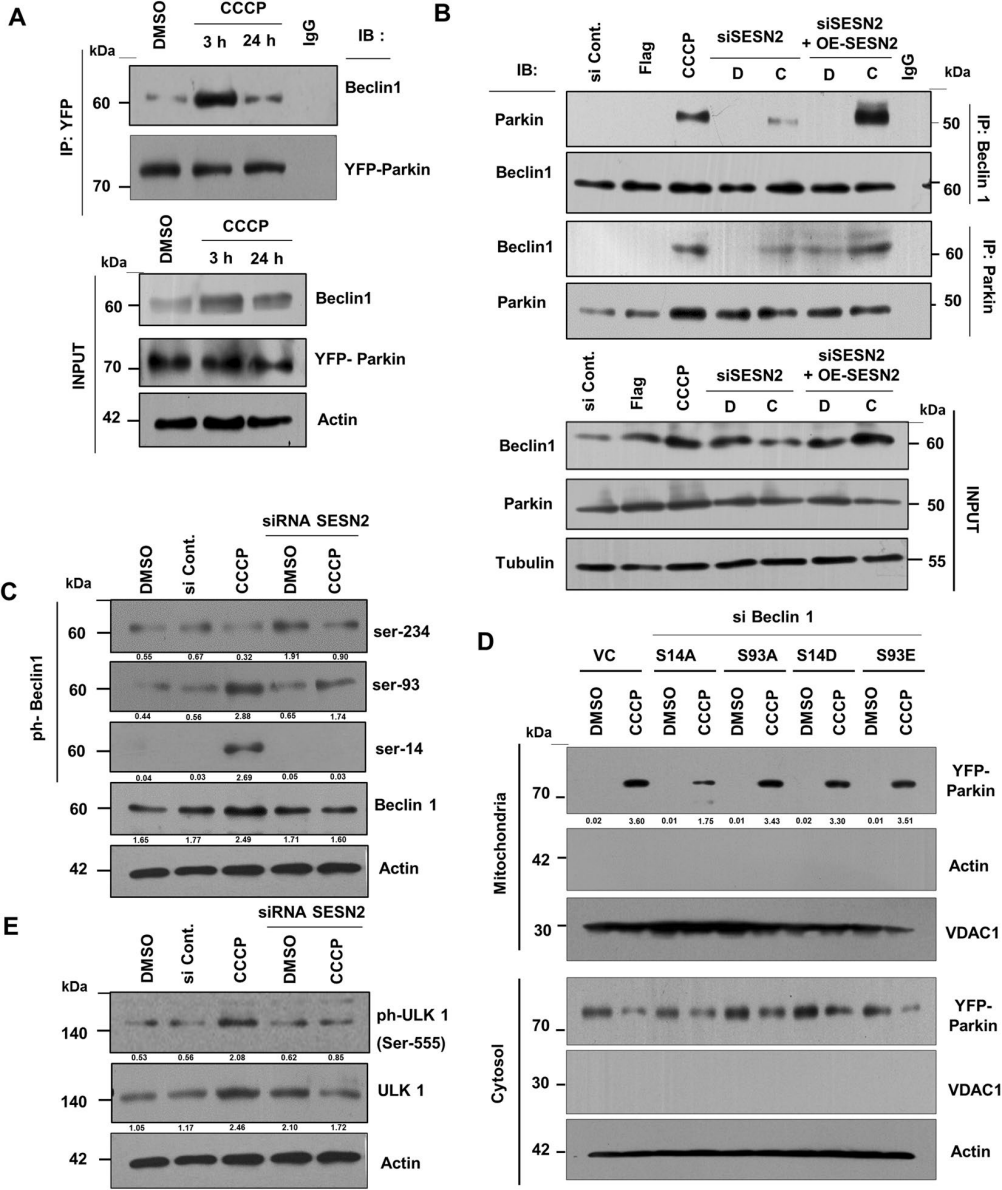
SESN2 facilitates Beclin1 and Parkin interaction during CCCP-induced mitophagy. (**A**) HeLa cells stably expressing YFP-tagged Parkin were treated with DMSO vehicle and CCCP for 3 h and 24 h and whole cell lysates were subjected to co-immunoprecipitation assay using anti-YFP antibody, for the detection of interaction between Beclin1 and Parkin. (**B**) Cells were transfected with scrambled siRNA, FLAG tagged empty plasmid, siRNA SESN2 and FLAG-tagged SESN2 overexpression plasmid and treated with CCCP for 3 h. Endogenous interaction of Parkin and Beclin1 was investigated by performing co-immunoprecipitation assay using antibodies against Parkin and Beclin1. 1-DMSO, 2-CCCP (3 h). A significant loss of interaction was observed on knockdown of SESN2 (si SESN2) which restored on overexpression of the same (OE-SESN2). (**C**) HEK293T cells were transfected with scrambled siRNA and siRNA targeting SESN2 and treated with DMSO (vehicle) and 10 µM CCCP for 3 h. Subsequently, western blotting was performed and phosphorylation of Beclin1 at serine-234, serine-93, and serine-14 positions was detected with relevant antibodies. Relative density (protein/Actin) protein bands has been shown in figure as numerical values. (**D**) HeLa cells stably expressing YFP tagged Parkin were transfected with siRNA targeting Beclin1, plasmid expressing phospho-mutants of Beclin1 for serine 93 (S93A, S93E) and serine-14 (S14A, S14D) positions. Mitochondrial and cytosolic fractions were isolated and analysed for YFP-Parkin mitochondrial accumulation and relative density (Mitochondria/Cytosol) of YFP-parkin protein bands was shown in figure. VDAC1, mitochondrial marker and ACTB/Actin, cytosolic marker. (**E**) Variation in the level of ULK1 and its phosphorylation at serine-555 position was detected by immunobloting. 10 µM CCCP for 3 h. Relative density (protein/Actin) protein bands has been shown in figure. Immunoblots were converted to gray and white scale using Adobe Photoshop CS6, with linear adjustment of exposure or contrast was done as per required. Images of immunoblots were cropped for clarity, expanded view of Fig. 4C, D and E is available in supplementary figures.

Having established the mechanism of activation of SESN2, we sought to understand the mechanism of SESN2 activation regulating the mitochondrial translocation of Parkin. Prior studies show that Beclin1 interacts with Parkin and promotes autophagy^27^, but it is not known if SESN2 has any role in regulation of this interaction. Co-immunopreciptation studies were carried out in HeLa cells stably expressing YFP-Parkin and a significant increase in the interaction was observed at 3 h of CCCP treatment with a subsequent decline at 24 h (Fig. 4A). Downregulation of SESN2 by siRNA during CCCP treatment resulted in a loss of interaction between endogenous Beclin1 with Parkin but overexpression of SESN2 facilitated the interaction (Fig. 4B). This suggested an important role of SESN2 in the regulation of Beclin1 and Parkin interaction but the question was how SESN2 promoted this association during CCCP-induced mitophagy. Since, Beclin1 phosphorylation is essential for regulation of the various cellular functions^28,29^, delving a little deeper in the process of Beclin1 binding to Parkin and its possible dependence on SESN2, we explored the phosphorylation status of Beclin1 in wild-type and SESN2 downregulated cells. It is pertinent to mention that ULK1 (ATG1) mediated Beclin1 phosphorylation at serine-14 position is crucial for autophagy induction^30^. The experiments showed a significant reduction in the phosphorylation of Beclin1 at serine-14 and serine-93 position on SESN2 downregulation (Fig. 4C), suggesting that SESN2 is essential for phosphorylation of Beclin1. Further, to evaluate the role of respective phosphorylation in regulation of the Parkin translocation, analysis of mitochondrial and cytosolic fractions were done in cells transfected with phospho-deficient and phospho-mimetic mutants of Beclin1 for serine-14 and 93 positions respectively. Results showed that only phospho-deficient mutation (S14A) of serine-14 position but not 93 of Beclin1, was able to reduce the accumulation of Parkin to damaged mitochondria (Fig. 4D). Interestingly, the phosphomimetic S14D mutants were able to assist Parkin translocation to the mitochondria only on CCCP treatment highlighting the necessity of the signals generated from damaged mitochondria (Fig. 4D). Furthermore, reduction in phosphorylation of ULK1 (serine-555) was observed on downregulation of SESN2 expression which suggest that SESN2 regulates the activation of ULK1 (Fig. 4E). These results suggest SESN2 mediated activation of Beclin1 through ULK1 mediated phosphorylation could be responsible for Beclin1 and Parkin interaction eventually leading to Parkin translocation to damaged mitochondria.

### SESN2 interacts with ULK1 and promotes phosphorylation of Beclin1 during CCCP-induced mitophagy

**Figure 5:**
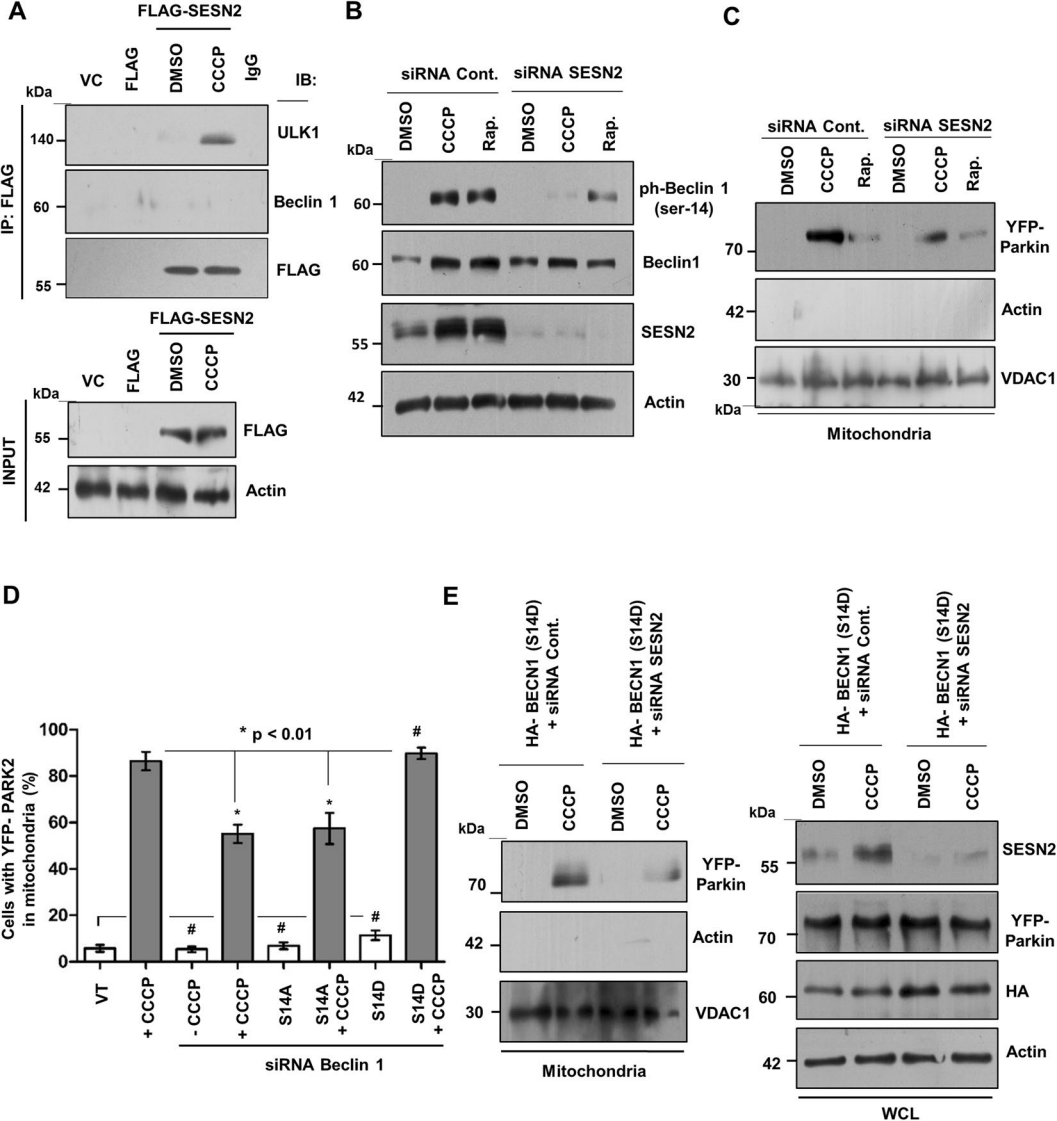
SESN2 interacts with ULK1 on CCCP-induced mitophagy and assists migration of Parkin. (**A**) HEK293T cells were co-transfected with empty FLAG tagged plasmid and plasmid expressing FLAG tagged SESN2 and subsequently treated with DMSO and CCCP for 3 h. Total cell lysates were subjected to co-immunoprecipitation using anti-FLAG M2 magnetic beads. Western blot analysis shows that SESN2 interacts with ULK1 on CCCP treatment but not with Beclin1. (**B**) Wild-type and SESN2 knockdown cells were treated with 10 µM CCCP and 500 nM Rapamycin for 3 h and ULK1 mediated serine-14 phosphorylation of Beclin1 was analysed by immunoblotting. (**C**) HeLa cells stably expressing YFP-tagged Parkin, which were transfected with scrambled siRNA and siRNA targeting SESN2 and treated with CCCP and rapamycin as per mentioned above. Mitochondria was isolated and western blot analysis was done to determine the amount of YFP-Parkin in mitochondria. Note: reduction in Parkin mitochondrial accumulation was observed only in CCCP treated SESN2 knockdown cells. VDAC1, mitochondrial marker and ACTB/Actin, cytosolic marker. (**D**) HeLa cells stably expressing YFP-Parkin were co-transfected with siRNA targeting Beclin1, HA-tagged phospho-mutant Beclin1 S14A, HA-tagged phospho-mimetic S14D and subsequently treated with 10 µM CCCP (3 h). YFP-Parkin co-localization with TOMM20 in HeLa cells stably expressing YFP-Parkin was scored after counting > 100 cells per sample in three independent experiments. The error bars in graph represents mean ± SEM. Parkin translocation is inhibited in cells with phospho-deficient mutation (S14A) while translocation occurs only on CCCP treatment with the phosphomimetic mutant (S14D). S14A, phospho-deficient mutation; S14D, phosphomimetic mutant. *p < 0.01, # NS. (**E**) HeLa cells stably expressing YFP-Parkin were co-transfected with scrambled siRNA, siRNA targeting SESN2 and HA-tagged phospho-mimetic S14D of Beclin1. Western blot analysis of mitochondrial fraction and whole cell lysates was done. VDAC1, mitochondrial marker and Actin, cytosolic marker. 10 µM CCCP for 3 h. Immunoblots were converted to gray and white scale using Adobe Photoshop CS6, with linear adjustment of exposure or contrast. Images of immunoblots were cropped for clarity, expanded view of Fig. 5A and E is available in supplementary figures.

Having established that knockdown of SESN2 reduces ULK1 activity, we checked if SESN2 directly controls ULK1. Interaction of FLAG-SESN2 with endogenous ULK1 was determined and the results demonstrate interaction of SESN2 with ULK1 with major interaction occurring upon CCCP-induced damage. Conceivably, this interaction may cause conformational change in ULK1, thereby enhancing ULK1 activity (Fig. 5A). Next, we explored whether SESN2 depletion blocks ULK1 mediated Beclin1 (ser-14) phosphorylation during mTOR dependent autophagy. To explore this, we analysed the levels of SESN2 under mTOR inactivation (using Rapamycin and Evrelimous) and CCCP treatment. The level of SESN2 significantly increased on both mTOR inactivation and CCCP treatment confirming SESN2 as a stress inducible gene (Fig. S3D). Further, to understand if SESN2 mediated ULK1 dependent Beclin1 phosphorylation was specific to CCCP-induced mitochondrial damage or not, we checked phosphorylation of Beclin1 (serine-14) under CCCP and rapamycin treatment in wild type and SESN2 downregulated cells. The results demonstrated that the phosphorylation of Beclin1 occurs under both rapamycin and CCCP treatment. However, a marked reduction in the phosphorylation of Beclin1 was obtained in SESN2 knockdown cells post CCCP treatment whereas a slight reduction was noted under mTOR inhibition. This feeble decline in phosphorylation under mTOR inhibition in SESN2 downregulated cells could be due to either decline in the level of Beclin1 or inhibition of macroautophagy (Fig. 5B). Notably, the mitochondrial translocation of Parkin did not decline in rapamycin treated SESN2 downregulated cells suggesting that SESN2 mediated regulation of Parkin migration was specific to CCCP-induced mitochondrial damage (Fig. 5C). Next, to confirm if serine-14 phosphorylation of Beclin1 was sufficient for Parkin translocation, HeLa cells stably expressing YFP-tagged Parkin were transfected with Beclin1 (S14A) and Beclin1 (S14D) phospho-mutants (S14A as non-mimetic and S14D as mimetic). Microscopic observations suggested a reduced translocation of Parkin in S14A mutant expressing cells even after CCCP treatment. However, S14D mutant expressing cells showed competent translocation of Parkin to mitochondria upon CCCP-induced stress (Fig. 5D). These results were further confirmed by analysis of the mitochondrial fraction for Parkin where no significant translocation of Parkin was observed in untreated S14D mutants, but migration robustly increased on CCCP treatment (Fig. S3E), indicating CCCP induced depolarization is obligatory for translocation of Parkin to mitochondria. Further, if SESN2 regulates Parkin translocation to mitochondria by increasing Beclin1 (serine-14) phosphorylation (presumably by interacting with and upregulating ULK1 activity), then the expression of the Beclin1 phospho-mimetic (S14D) mutant should restore the Parkin translocation. Results suggested that expression of S14D mutant was able to partly restore the Parkin migration in SESN2 knockdown cells on CCCP treatment implicating the role of other regulators in Parkin translocation (Fig. 5E). In summary, our findings suggest that SESN2 promotes the interaction of Parkin and Beclin1 by inducing ULK1 activity and thereby mediates phosphorylation of Beclin1 (serine-14) following damage to mitochondria.

### PINK1 is indispensable for mitochondrial translocation of Parkin but not for its interaction with Beclin1

**Figure 6:**
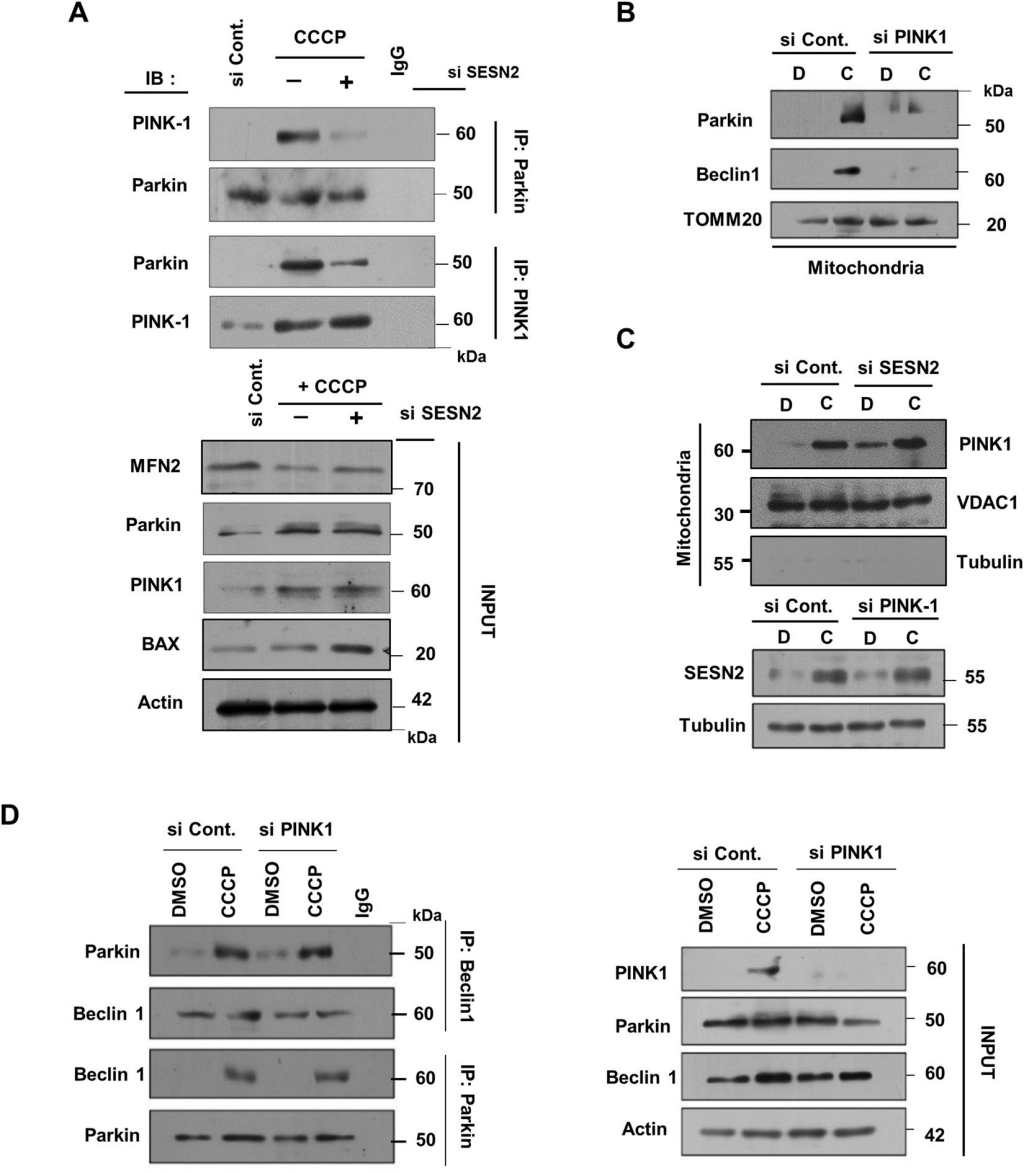
PINK1 is required for Parkin translocation but interaction between Beclin1 and Parkin is PINK1 independent. (**A**) HEK293T cells were transfected with scrambled siRNA and siRNA targeting SESN2 followed by treatment with 10 µM CCCP for 3 h. Total cell lysates were co-immunoprecipitated using anti-PINK1 and anti-Parkin antibodies, to find the variation in interaction between endogenous PINK1 and Parkin. Interaction of PINK1 with Parkin is less prominent on downregulation of SESN2. (**B**) Effect of PINK1 on mitochondrial translocation of Parkin was assessed by transfecting cells with scrambled siRNA and PINK1 siRNA followed by treatment with 10 µM CCCP for 3 h. Cellular sub-fractionation was done and mitochondrial fraction was subjected to immunoblotting. TOMM20, mitochondrial marker. 1-DMSO, 2-CCCP. Note that PINK1 knockdown abolishes the presence of Parkin. (**C**) To check the mitochondrial stabilization of PINK1 in SESN2 knockdown cells, cells were treated with DMSO (vehicle) and CCCP for 3 h and western blot analysis was carried out in isolated mitochondrial fractions. VDAC1, mitochondrial marker and TUBB/β-Tubulin, cytosolic marker. Levels of SESN2 in PINK1 knockdown cells were analysed with western blots in whole cell lysates. 1-DMSO, 2-CCCP. Note: downregulation of SESN2 or PINK1 was unable to decline stability of each other. (**D**) Wild-type (WT) and PINK1 knockdown cells were treated with DMSO (vehicle) and CCCP for 3 h. Cytosolic fractions were isolated and subjected to co-IP assay using anti-Beclin1 and anti-Parkin antibodies. Immunoblots were converted to gray and white scale using Adobe Photoshop CS6, with linear adjustment of exposure or contrast. Images of immunoblots were cropped for clarity, expanded view of Fig. 6D is available in supplementary figures.

**Figure 7:**
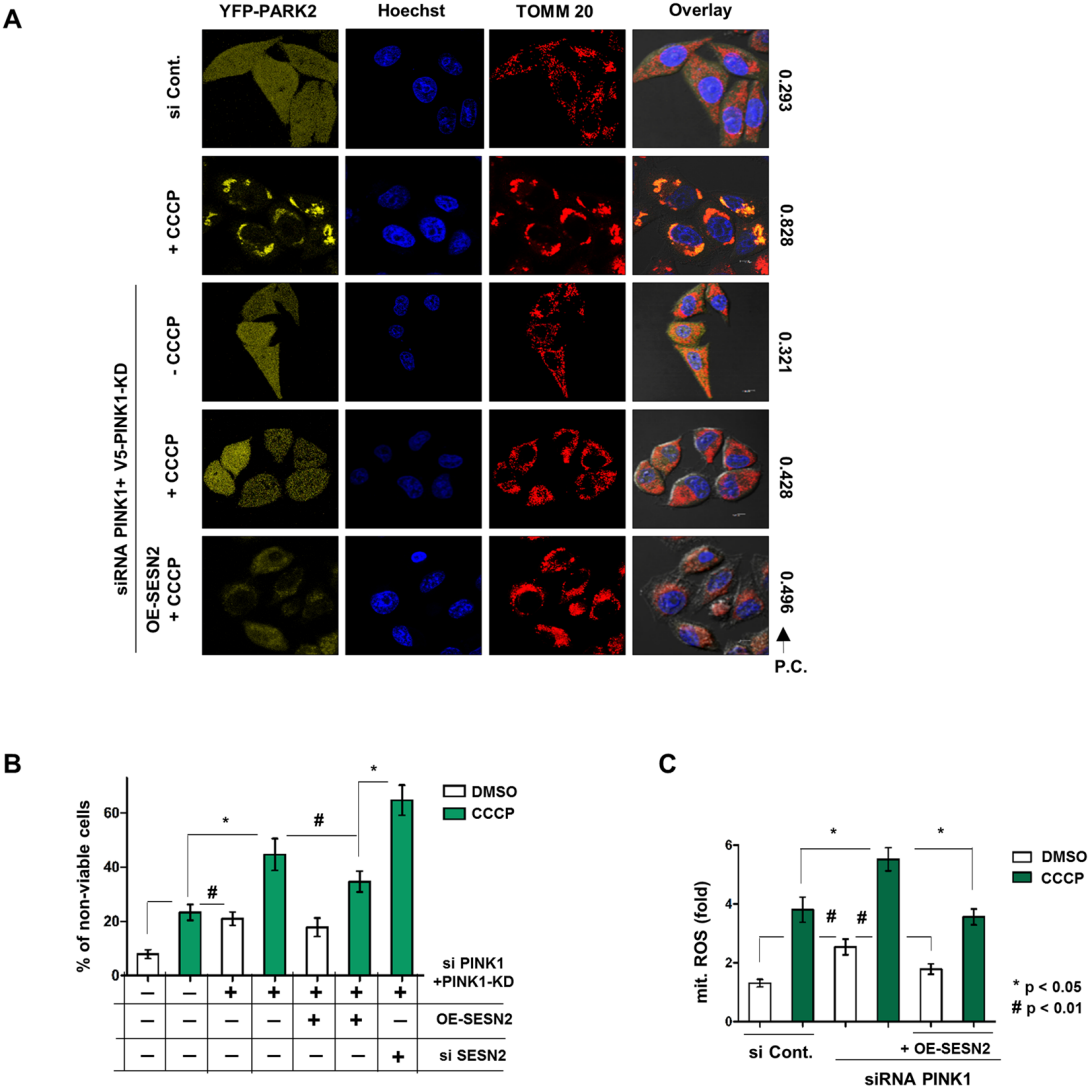
Overexpression of SESN2 rescue from CCCP-induced damage in PINK1 downregulated cells but not Parkin mitochondrial translocation. (**A**) HeLa cells stably expressing YFP-Parkin were transfected with scrambled siRNA, siRNA PINK1 along with plasmid expressing kinase dead domain mutant of PINK1 and treated with 10 µM CCCP for 3 h. To investigate the mitochondrial translocation of Parkin, cells were immunostained using antibody against TOMM 20. To evaluate if SESN2 can rescue the effect of PINK1 knockdown, plasmid expressing SESN2 was transfected. Scale bars, 10 µm. (**B**) Cells were transfected with siRNA targeting SESN2 and PINK1, plasmids expressing PINK1 kinase dead domain mutant and SESN2. Cell viability was determined by MTT assay on DMSO or CCCP treatment (12 h) as indicated. The error bars represents mean ± SEM from three independent experiments (n = 3). It is notable that overexpression of SESN2 was able to protect cells from CCCP induced damage in PINK1 knockdown cells. *p < 0.01, #p < 0.05 C. Evaluation of mitochondria generated superoxide was done by mitoSOX assay in cells which were transfected with siRNA targeting PINK1 and overexpression plasmid of SESN2 followed by treatment with DMSO and CCCP (12 h). The error bars represent mean ± SEM (n = 3). The figure shows overexpression of SESN2 was able to decline increase in CCCP induced generation of mitochondrial ROS in PINK1 knockdown cells. *p < 0.01, #p < 0.05.

Requirement of PINK1 in translocation of Parkin to damaged mitochondria is the central act in mitophagy^7,31^. We explored whether SESN2 can compensate for the requirement of PINK1 for translocation of Parkin to the damaged mitochondria during mitophagy. Results demonstrated that SESN2 downregulation during 3 h CCCP treatment results in a substantial inhibition of PINK1 and Parkin interaction (Fig. 6A) clearly suggesting the role SESN2 in the interaction of PINK1 and Parkin. Prior literature suggests a PINK1 dependence of mitochondrial translocation of Parkin in response to depolarization^26,32^. When PINK1 was downregulated using siRNA, both Parkin and Beclin1 were not detected in the mitochondrial fraction after CCCP stress (Fig. 6B), suggesting that PINK1 is required for Parkin translocation to the damaged mitochondria. To understand if inhibition of Parkin migration to mitochondria on SESN2 downregulation is due to the decline in outer mitochondrial membrane (OMM) stabilization of PINK1, we downregulated SESN2 and observed an increase in PINK1 stabilization on damaged OMM suggesting that SESN2 regulates mitochondrial translocation of Parkin without affecting stabilization of PINK1. Conversely, PINK1 downregulation does not affect levels of SESN2 thereby suggesting that SESN2 and PINK1 both regulate the mitochondrial translocation of Parkin without affecting stability of each other (Figs 6C and S4A and B). Further, PINK1 downregulation did not disrupt the Parkin and Beclin1 interaction (Fig. 6D) suggesting SESN2 mediated Parkin-Beclin1 interaction was PINK1 independent. Prior knowledge of the requirement of PINK1 kinase activity for Parkin translocation^8^ prompted us to express a PINK1 kinase dead domain mutant and check the translocation of Parkin. Consistent with observations from other reports^26^, cells expressing PINK1 kinase dead domain mutant did not show presence of Parkin in mitochondrial fractions. However, overexpression of wild-type PINK1 harboring kinase domain enabled Parkin to translocate (Fig. S4C). This was evidence of absolute requirement of the kinase activity of PINK1 for mitochondrial translocation of Parkin. Microscopic evidence corroborated the western blot data and also showed that SESN2 overexpression failed to rescue translocation of YFP-Parkin when kinase activity of PINK1 was abolished (Figs 7A and S4D). Further, experiments indicated that overexpression of SESN2 prevents cell death in CCCP-induced cellular damage in PINK1 downregulated cells, defining cytoprotective role of SESN2 (Fig. 7B). Analysis of generation of mitochondrial ROS demonstrated that overexpression of SESN2 curbed the increase in mitochondria generated ROS on PINK1 downregulation (Fig. 7C).

## Discussion

Sestrins are stress sensor proteins having multiple roles in cellular homeostasis^24^. They are involved in autophagy^13^, however, their role in mitophagy, a selective form of mitochondrial autophagy remains less understood. The clue to a possible role of Sestrins in mitophagy came from studies with *Drosophila melanogaster* where deletion of the dSesn gene resulted in accumulation of dysfunctional mitochondria leading to age related pathologies^18^. Recent studies also have suggested SESN2 involvement in mitophagy^12,22^. The current study demonstrates a novel role of SESN2 in the regulation of mitophagy in mammalian cells. The pivotal event in mitophagy is the translocation of cytosolic Parkin, an E3 ubiquitin ligase from the cytosol to the mitochondria and binding to the mitochondrial outer surface protein PINK1, a serine/threonine-protein kinase^10^. To demonstrate if SESN2 was involved in this translocation process, we used the model of CCCP induced Parkin translocation in HEK293T cells^32^, where downregulation of SESN2 resulted in significantly diminished Parkin translocation. However, overexpression of SESN2 reversed this reduction implying SESN2 participation in the process. These observations of activation of SESN2 and its involvement in Parkin translocation was similar when other mitochondria specific damaging agents (oligomycin + antimycin A) were used. The functional importance of Parkin translocation for CCCP-induced mitophagy has been earlier established^7^. Our observations of increase in cell death and reduction in mitochondrial translocation of Parkin on SESN2 downregulation upon CCCP as well as oligomycin/antimycin A treatment suggested the importance of SESN2 in Parkin mediated mitophagy and cell survival.

The post translocation binding of Parkin to PINK1 on the mitochondrial surface triggers dismantling of the damaged organelle and the success of mitophagy is dependent on this event^9^. In addition to a competent PINK1, another required event for Parkin translocation is its binding to Beclin1^27,33^. Using Alzheimer’s disease (AD) animal models it has been shown that interaction of Beclin1 with Parkin contributes to clearance of amyloid aggregates^27^. Beclin1, a tumor suppressor protein, has multiple binding partners and is an important regulator of cellular function^34^. Our observation of a reduced interaction between Parkin and Beclin1 on SESN2 knockdown suggests SESN2 may be directly affecting either Beclin1 or Parkin in some way. Prior knowledge on the requirement of phosphorylation of Beclin1 as a prerequisite to interact with other binding partners^28,30^ led us to explore if phosphorylation of Beclin1 was dependent on SESN2. We detected reduced phosphorylation of Beclin1 at serine-14 and 93 positions when SESN2 was downregulated. This observation was important because, Beclin1 phosphorylation at both ser-14 and 93 positions is crucial for regulation of autophagy^28–30^. However, our analysis showed that only phospho-deficient mutation (S14A) of serine-14 but not serine-93 position of Beclin1 resulted in reduction of the accumulation of Parkin to the damaged mitochondria. The question was how did SESN2 regulate Beclin1 phosphorylation. Based on existing information regarding Beclin1 phosphorylation by ULK1 kinase^29,30^, we checked if SESN2 was involved in the activation of ULK1. Evidence for a significant involvement of SESN2 came from experiments where SESN2 downregulation was shown to interfere with ULK1 phosphorylation at ser-555, required for its activity. The SESN2 interacts with ULK1 and promotes autophagic degradation of p62 associated targets but its regulatory role in mitophagy has never been defined. We demonstrate that for efficient mitochondrial translocation of Parkin during CCCP induced mitophagy, SESN2 interacts with ULK1 and induces ULK1 mediated phosphorylation of Beclin1 (serine-14) which facilitates mitochondrial localization of Parkin. Taken together, the above data suggest SESN2 to be a crucial regulator of mitochondrial translocation of Parkin in CCCP- induced mitophagy.

Damaged mitochondria emanate signals for translocation of Parkin and mitophagy^35–38^. In the backdrop of SESN2 involvement in Parkin and Beclin1 binding, it was of interest to see if this was triggered by signals generated by the mitochondria with impaired potential (∆Ψ_m_). Since mimetic phosphomutants were able to assist Parkin to translocate to the mitochondria only on CCCP treatment but failure of translocation without CCCP exposure confirmed the notion that damaged mitochondria provided required signals. CCCP treatment often leads to mitochondrial superoxide generation^39^ but also has been reported to decline the same^40^ and this could be the associated signal for Parkin translocation. It is known that mitochondrial redox state is associated with defective mitophagy^38^ and since the SESN2 is associated with oxidative stress, it suggests a possible role of SESN2 in maintaining the mitochondrial redox state. Observations in our study show that selective scavenging of mitochondrial superoxide being able to inhibit SESN2 activation and mitochondrial translocation of Parkin. How SESN2 selectively senses mitochondria generated superoxide still remains elusive. It may be possibly linked with formation of mitochondrial permeability transition pore but further experimental studies are needed to establish this concept.

It was important to check if this role of SESN2 was specific for CCCP induced stress or also occurred during rapamycin induced autophagy. During mTOR dependent autophagy where mTOR was inactivated through rapamycin treatment, SESN2 downregulation did not reduce Parkin translocation as compared to CCCP treatment, suggesting that SESN2 mediated regulation of Parkin migration was specific to CCCP-induced mitochondrial damage involving uncoupling of the proton gradient.

**Figure 8:**
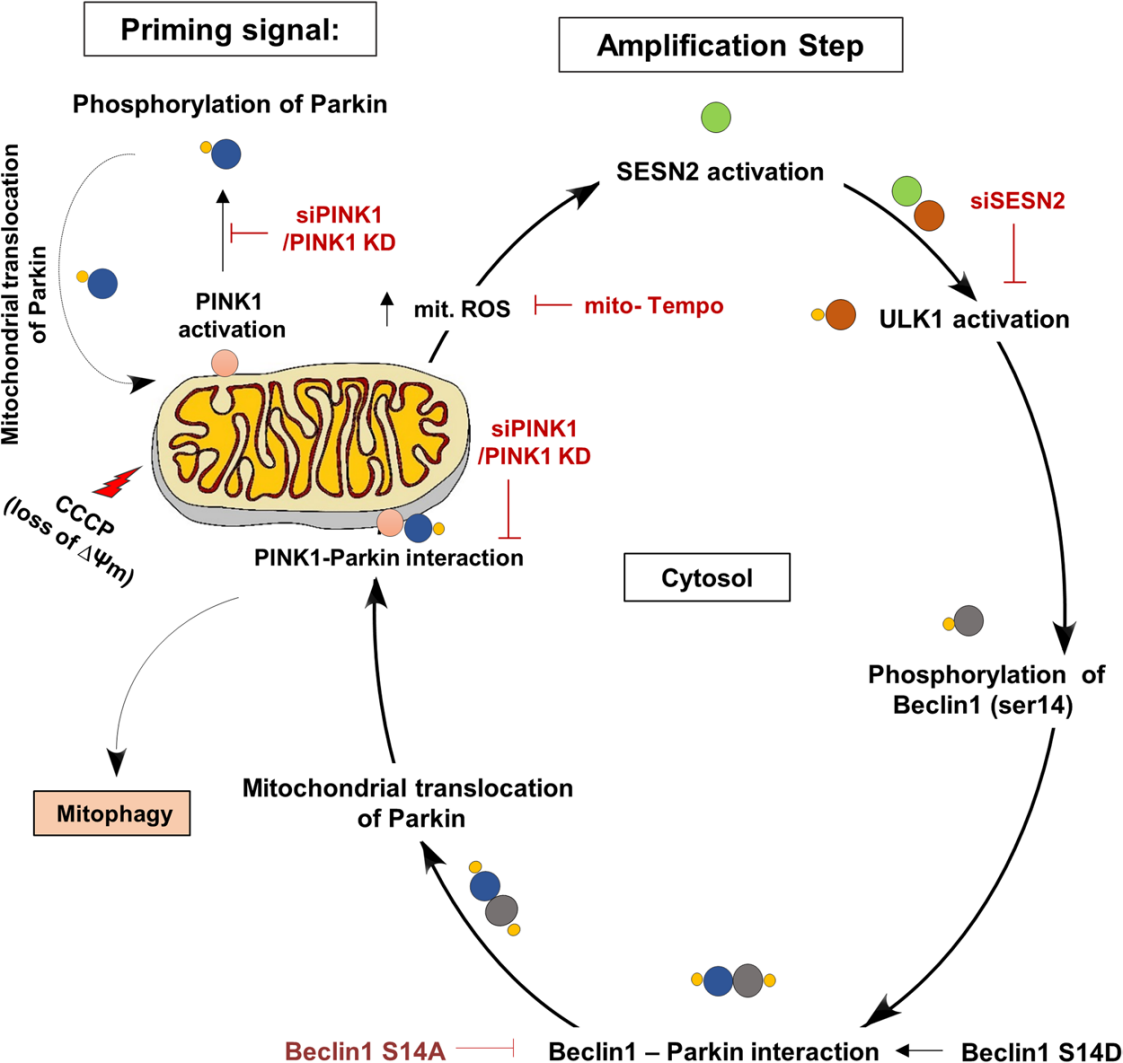
Schematic representation of proposed model of the study. The two step working model defining the role of SESN2 in amplification step of Parkin translocation on damaged mitochondria during CCCP-induced mitophagy. The priming signal generated by phosphorylation of Parkin by PINK1 which is indispensable for Parkin translocation to mitochondria. During amplification step, SESN2 a stress inducible protein senses CCCP-induced increase in mitochondria generated superoxide which facilitates mitochondrial accumulation of Parkin. Post activation of SESN2, SESN2 interacts with ULK1 which affects its activation, hence resulting in phosphorylation of Beclin1 at its serine14 position. Phosphorylation of Beclin1 results in its interaction with Parkin which facilitates Parkin mitochondrial translocation. Notably, the complex formation between Beclin1 and Parkin is independent of priming signal generated by PINK1 but requires priming signal for translocation to mitochondria.

The requirement of PINK1 and its kinase activity has been shown to be indispensable for the mitochondrial translocation of Parkin and mitophagy^32^. Our data show that SESN2 overexpression was unable to recompense Parkin translocation to the damaged mitochondria in cells where PINK1 was either downregulated or PINK1 kinase was inactive, attesting the obligatory nature of PINK1 in the process. Although PINK1 initiates the Parkin translocation but downregulation of SESN2 supresses the rate of translocation of Parkin, it emphasises the requirement SESN2 in the process. The SESN2 mediated Beclin1-Parkin complex formation remains unaffected on PINK1 knockdown but mitochondrial translocation of Parkin was abolished (Fig. 8). Based on our findings, a two-step mechanistic model could be proposed where PINK1 mediates priming for translocation of Parkin and SESN2 amplifies the process by forming Beclin1-Parkin complex which is independent of PINK1. Activation of SESN2 in response to mitochondrial generated superoxide may promote its interaction with ULK1 which is crucial for downstream signalling for Parkin mitochondrial translocation. Henceforth, our novel findings open an avenue for exploration of additional mechanistic roles of SESN2 in regulation of quality check of mitochondria especially where mitochondrial integrity is affected.

## Materials and Methods

### Media and reagents

Anti-Flag M2 magnetic beads were purchased from Sigma-Aldrich (M-8823), 10% heat inactivated Fetal Bovine Serum (Biological Industries, 04-127-1 A), Mito-Tempo (Enzo Life Sciences, ALX-430-150-M005), CBX protein assay kit (G-biosciences, 786-12 ×), FemtoLucent ECL kit (G-biosciences, 786-056), Lipofectamine LTX-3000 (Invitrogen, L3000015), Dynabeads Protein G (1000D) and Dynabeads® Immunoprecipitation Kit (10007D) from Thermofisher Scientific. Ni-NTA beads (30210) and Qproteome mitochondrial isolation kit (37612) were purchased from Qiagen. Nitrocellulose membrane (HATF00010) was purchased from Merck Millipore Ltd. Unless otherwise mentioned all the chemicals used for experiments were purchased from Sigma-Aldrich.

### Plasmids and siRNA

The siRNA #1 silencer select targeting SESN1 (S26030) and SESN2 (S38099) were purchased from Ambion, siRNA #2 targeting SESN1 (SR309047) and SESN2 (SR313205) were purchased from Origene, siRNA #3 (on-Target *plus* smartpool, which is mixture of 4 siRNAs in single reagent) targeting SESN1(L-020244-00) and SESN2 (L-019134-02) were purchased from GE Dharmacon. The siRNA targeting PINK1 (SR312201) and siRNA BECN1/Beclin1 (SR305711) were synthesized from Origene. FLAG-tagged SESN2, myc-tagged PINK1, V5- tagged PINK1 kinase dead (KD), HA-BECN1/Beclin1 (S14A) and HA-BECN1/Beclin1 (S14D), pRK5-myc-Parkin, pCHAC-mt-mkeima and pclbw-mitoTag RFP plasmids were purchased from Addgene. Phosphomutants of Beclin1 (S93A and S93E) were kind gift from Dr. Richard J. Youle (NINDS, Bethesda MD, USA). HA-pCDNA, FLAG-pCDNA plasmids were obtained from Dr. A.C. Banerjea (NII, New Delhi, India).

### Antibodies

Anti-PINK1 (Novus, BC100-494), anti-Actin (A2668) and anti-FLAG (F3165) from Sigma-Aldrich, anti-β-Tubulin (Thermofisher, PA1-41331), anti-SESN2 (Proteintech, 10795-1-AP, Santa Cruz, SC-101249), anti-HA (BD biosciences, 552565), anti-TOMM20 (BD biosciences, 612278), anti-BECN1/Beclin1 (BD biosciences, 612113), anti-FLAG (Sigma, F3165), anti-GFP/YFP (Abcam, ab290), anti-BAX (2272), anti-ULK1 (8054), anti-MFN2 (9482), anti-parkin (4211), anti-phospho ULK1 (Ser555) (5869), anti-phospho BECN1/Beclin1 for serine-14 (84966) and serine-93 (14717) were purchased from cell signaling technology (CST) and for serine-234 (ab-183335) was purchased from Abcam. Antibody against SESN1 was a generous gift from Dr. Jun Hee Lee (University of Michigan, Ann Arbor, USA).

### Cell Culture

HeLa, HEK293T and SH-SY5Y cell lines were purchased from ATCC. HeLa cells stably expressing YFP tagged Parkin was a kind gift from Dr. Richard J. Youle (NINDS, Bethesda MD, USA). HeLa and HEK293T cells were maintained in Dulbecco’s modified Eagle’s medium (Sigma Aldrich, D6046) where as SH-SY5Y cells were maintained in DMEM-F12 media (Gibco®/BRL, Life Technologies, 11320-033) with 10% heat-inactivated fetal bovine serum in 5% CO_2_ chamber. To induce mitochondrial depolarization, cells were treated with 10 μM carbonyl cyanide m-chlorophenylhydrazone (CCCP).

### Transfection

For siRNA transfections, low passage cells were transfected using n-Ter peptide transfection kit (Sigma Aldrich, N2913). To achieve efficient knockdown of gene, post 36 h of first transfection, cells were again transfected using same method and cells with ≥ 90% downregulation efficiency were used for experimental setup^26^. For transfection of plasmids, cells were seeded 24 h prior transfection and were transfected using lipofectamine LTX at a concentration of 2% of cultured media.

### Western Blotting

Whole cell lysates were prepared using 1 × sample buffer containing 2% SDS, 10% glycerol, 0.1% 2-Mercaptoethanol and 100 Mm Tris-Cl (pH 6.8) and estimation of proteins was done using CBX assay kit. Equal amount of protein samples were loaded and resolved on 12% SDS-PAGE gel and subsequently transferred onto nitrocellulose membrane as described previously^41^. Further, transblotted proteins were blocked using 5% Bovine Albumin Serum (BSA) and immunoblotting was done using relevant primary and secondary antibodies. Analysis of results was done with enhanced chemiluminescence (ECL) using FemtoLucent kit and autoradiography using x-ray film. Quantitation of density of signals on immunoblots was performed using GeneTools software (Syngene).

### Co-immunoprecipitation assay

Immunoprecipitation (IP) using antibodies for respective proteins: for co-immunoprecipitation of endogenous proteins, antibodies against Beclin1, PINK1, SESN2 and Parkin were used while for YFP-Parkin pulldown polyclonal anti-GFP was used. 5 × 10^7^ cells were transfected using required siRNA or plasmid constructs and lysed in lysis buffer containing 20 mM HEPES, 100 mM NaCl, 1% Triton X-100, 150 mM NaCl, 20 mM Tris (pH 7.5), 0.5 mM PMSF, 0.5 mM EDTA, and in the presence of protease inhibitors (Roche). After sonication and incubation (4 °C, 30 min), cell lysates were centrifuged and supernatants were collected (20 min, 15000 g, 4 °C). The concentration of lysates was measured using CBX assay kit and 800–1000 μg of lysate protein was used for Co-IP using the Dynabeads Protein G Immunoprecipitation kit following the protocol provided by manufacturer. IgG isotype antibodies were used as negative control.

### Subcellular Fractionation

Mitochondrial and cytosolic fractions were isolated from cells using a proteome mitochondria isolation kit following the protocol described previously^42^. Protein concentration of fractions was determined using CBX assay kit. The purity and loading of mitochondrial fractions was assessed by levels of mitochondrial protein Tom 20, VDAC1 or COX IV determined on western blot. Analysis of proteins was done using respective antibodies after performing SDS-PAGE and western blotting.

### Cellular staining and confocal microscopy

To determine the mitochondrial translocation of Parkin to mitochondria, HeLa cells stably expressing YFP tagged Parkin were given different treatments and its co-localization with TOMM20 was assessed by confocal microscopy. For immunostaining of mitochondria, cells were plated on poly-L-lysine coated slides and fixed using 4% paraformaldehyde. After blocking the cells with 3% BSA, cells were permeabilized using 0.05% Triton X-100 and the primary anti-mouse TOMM20 (1:100) was used to stain the mitochondria. For secondary antibodies, anti-Mouse-Alexa flour 594 (1:200) was used. Hoechst 33342 were used to stain nuclei. Slides were mounted using Vectashield (Vector Laboratories, H-1000) and imaged using a Leica TCS SP5II microscope (Wetlzar, Germany). Images were captured in point averaging mode at zoom value 3.0, using Plan Apo oil objective. Approximately, equal value of system optimized gain and offset values were used in all acquired images. Scattered dot plots with a defined Pearson coefficient for co-localization were generated. Events with value of Pearson coefficients above 0.7 were considered significant and were calculated only after removal of basal noise.

### Live cell imaging

Cells were transfected with mitochondria matrix targeted RFP (Red fluorescent protein) plasmid and post transfection sorted out for RFP signal using BD FACS Aria machine. Sorted cells were plated for microscopy in cell imaging dishes (Eppendorf, 0030740017) and images were captured as mentioned above. To measure mitophagy, LC3 puncta accumulation with mitochondria (mt-RFP) was done using live cell imaging.

### Mitophagy assay using mito-keima

HeLa cells stably expressing mito-Keima were generated as per previously suggested^26^. In brief, retroviruses were packaged in HEK293T cells post transfection with pCHAC-mt-mKeima-IRES-MCS2 plasmid. HeLa cells were transduced using collected viruses for 48 h with 6 µg/ml polybrene and fluorescence sorting was done to isolate cells stably expressing mito-Keima using BD FACS Aria cell sorter. Measurement of mitophagy was done by analysing mito-keima in lysosome by using dual excitation (458 nm and 560 nm) as per done previously^26^ and signal was acquired using filter-based multi-mode microplate reader (Clariostar, BMG Labtech, Germany).

### Measurement of cell viability and cell death

For analysis of cellular viability, MTT assay was performed as described earlier^43^. Cells were trypsinized and plated in 96-well plates overnight in incubator at 37 °C in 5% CO_2_. Transfected and non-transfected cells were treated with DMSO or CCCP respectively and 20 µl of 5 mg/ml tetrazolium dye MTT 3-(4,5-dimethylthiazol-2-yl)-2,5-diphenyltetrazolium bromide (Sigma-Aldrich, M5655) was added to each well. Following incubation for 4 h at 37 °C, supernatants were removed and 100 µl DMSO was added to dissolve precipitates. Absorbance was assessed at 590 nm using filter-based multi-mode microplate reader (Clariostar, BMG Labtech, Germany). Variation in the levels was represented as fold change as compared to the control.

Measurement of cell death using propidium iodide (PI) assay was performed as described previously^44^ and analysis was done by flow cytometry.

### Annexin and PI staining

Annexin/PI staining was done as per described earlier using dead cell apoptosis kit (Thermofisher scientific, V13242). To sort HeLa cells stably expressing YFP-Parkin, cells were stained with Annexin V-PE (phycoerythrin) conjugate (Thermofisher scientific, A35111) as per manufacturer’s instructions and sorting was done using BD FACS Aria machine.

### Measurement of mitochondrial ROS

Measurement of mitochondria generated ROS was carried out using mitoSOX red (molecular probes, M36008). Transfected and non-transfected cells were trypsinized, washed with 1 × PBS, centrifuged at 1000 × *g* for 5 min and resuspended using fresh media at density of 1 × 10^6^/ml. Subsequently, the cells were incubated with final concentration of 2.5* μ*M MitoSOX red for 30 min at 37 °C. Fluorescence emission of oxidized MitoSOX red was analysed by a filter-based multi-mode microplate reader (Clariostar, BMG Labtech, Germany). Variation in the levels was represented as fold change as compared to the control.

### Determination of mitochondrial membrane potential

Mitochondrial membrane potential (Ψ_m_) was determined using an anionic (lipophilic) mitochondrial JC-1 dye(5, 5′, 6,6′-tetrachloro1, 1′, 3, 3′tetraethylbenzimidozolylcarbocyanine iodide) (Invitrogen, T-3168) as described previously^41^. In brief, cells were washed using 1XPBS and incubated for 15 min at 37 °C with media containing 10 μM JC-1 dye. Emitted green (535 nm) and red (595 nm) fluorescence intensities were analyzed by filter-based multi-mode microplate reader (Clariostar, BMG Labtech, Germany) and ratiometric comparative fluorescence of the two were plotted using graph pad prism 5.1.

### Statistical Analysis

All experiments were independently repeated at least three times and represented as error ± SEM. To evaluate statistical difference and significance, an unpaired, two-tailed student’s *t*- test was performed. For all experiments p-value of ≤ 0.05 was considered statistically significant. Graphs were plotted using Graph pad prism 5.1.

## Electronic supplementary material

Additional Information
