# Supplementary material for: SESN2 facilitates mitophagy by helping Parkin translocation through ULK1 mediated Beclin1 phosphorylation
Source: Sci Rep. 2018 Jan 12;8:615. doi: 10.1038/s41598-017-19102-2 (PMC5766514; doi:10.1038/s41598-017-19102-2)
Supplement: Supplementary file 1 — Additional Information [file 41598_2017_19102_MOESM1_ESM.pdf]

SESN2 facilitates mitophagy by helping Parkin translocation through ULK1 mediated Beclin1 phosphorylation

Authors: Ashish Kumar[1], Chandrima Shaha[1*]

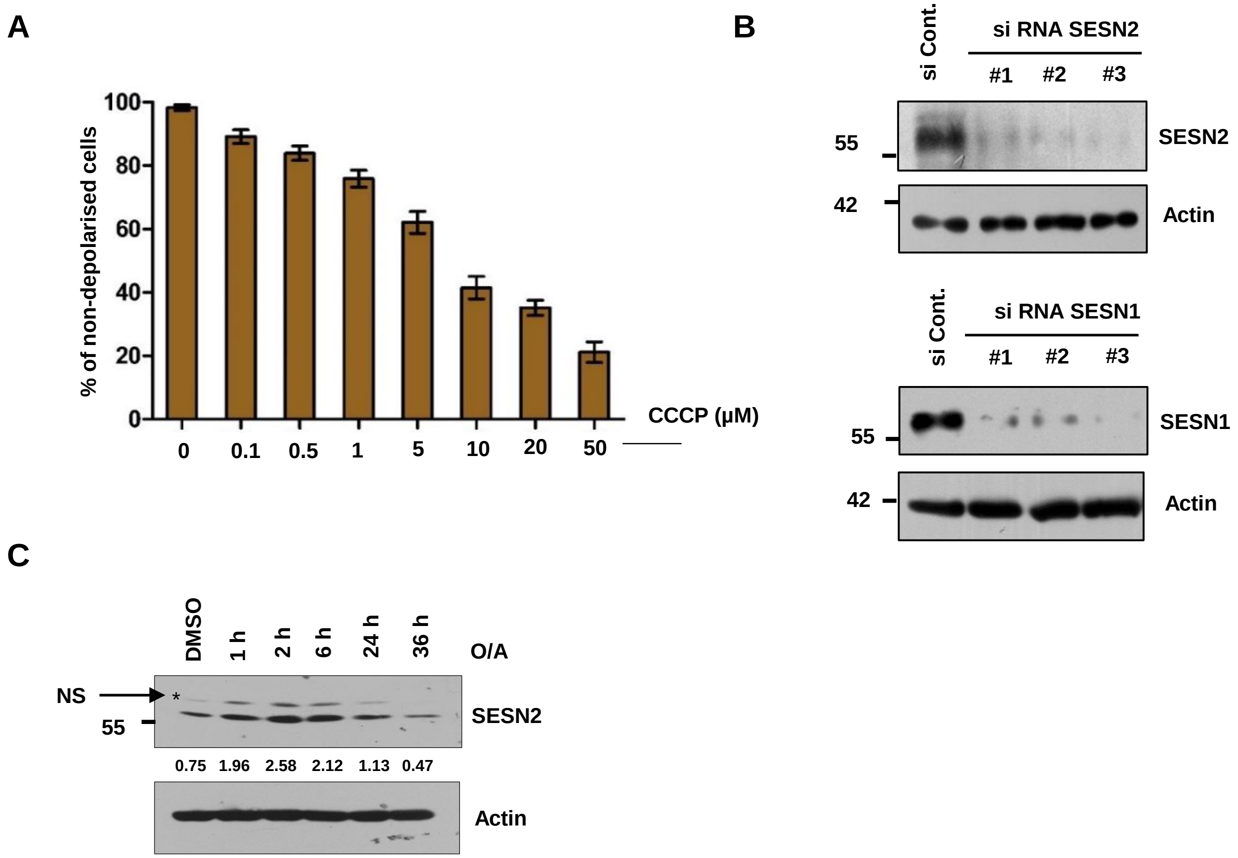

Fig S1

A. HEK293T cells were treated with CCCP in dose dependent manner (0.1 μM to 50 μM) for 3 h and percentage of cells with intact mitochondrial membrane potential ($\Delta\Psi_m$) was determined by JC-1 staining and detection of red to green signal ratio was scored by flourimetry. Error bars represent the mean ± SEM (n=3).

B. HEK293T cells transfected with scrambled siRNA and three different siRNA targeting SESN2 #1, #2, #3 and immunoblot analysis was done to measure percentage of downregulation of SESN2.

C. HEK293T cells were treated with 5 μM oligomycin + 5 μM antimycin A in time dependent manner and immuniblot analysis was done to analysis SESN2 level.

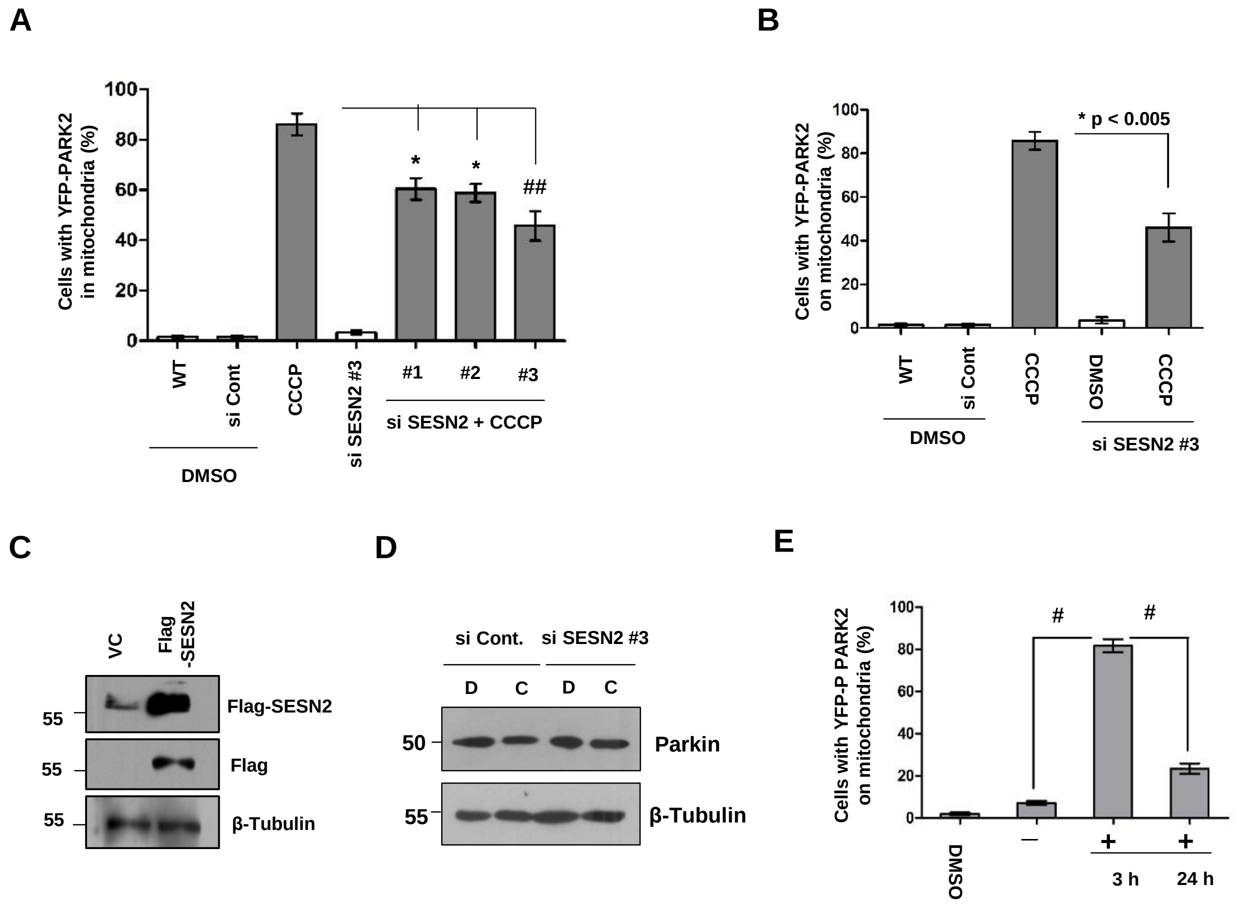

Fig.S2

A. HeLa cells stably expressing YF-tagged Parkin were transfected with scrambled siRNA and three different siRNA targeting SESN2 #1, #2, #3 and co-localization of YFP- Parkin with TOMM20 was scored after counting > 100 cells per condition in three independent experiments. CCCP treatment (3 h). Error bars represent the mean ± SEM (n=3). * $p < 0.01$. ## $p < 0.005$

B. HeLa cells stably expressing YFP-Parkin were transfected with scrambled siRNA and siRNA targeting SESN2 and treated with 10 µM CCCP for 3 h. Co-localization of YFP tagged Parkin with TOMM20 was scored after counting >100 cells per condition in three independent experiments. Error bars in the graph represents mean ± SEM (n=3). * $p < 0.005$

C. FLAG-tagged SESN2 was overexpressed in cells and immunoblot analysis was done to measure the percentage of overexpression.

D. Western Blot analysis of endogenous levels of Parkin in WT vs SESN2 downregulated HEK293T cells. D, DMSO; C, CCCP (3 h).

E. HeLa cells stably expressing YFP-Parkin were treated with CCCP for 3 h and 24 h. Co-localization of YFP tagged Parkin with TOMM20 was scored after counting >100 cells per condition in three independent experiments. Error bars in the graph represents mean ± SEM (n=3). # $p < 0.001$

F. HeLa cells stably expressing YFP-Parkin were transfected using scrambled siRNA and siRNA targeting SESN2 and treated with 10 µM CCCP for 3 and 24 h. mitochondrial translocation of Parkin was detected by co-localization with TOMM 20, immunostained using anti-TOMM 20. Further, for validation of results obtained, overexpression plasmid of FLAG-SESN2 was transfected in SESN2 knockdown cells. Pearson's coefficient (P.C.) > 0.7, significant. Scale bars, 10 µm.

G. Accumulation of LC3 puncta with mitochondria was observed and scored after counting >100 cells per condition in two independent experiments. Error bars represent the mean ± SEM (n=2). # $p < 0.05$ (compared to siCont. ,CCCP), ## $p < 0.005$ (compared to si Cont.).

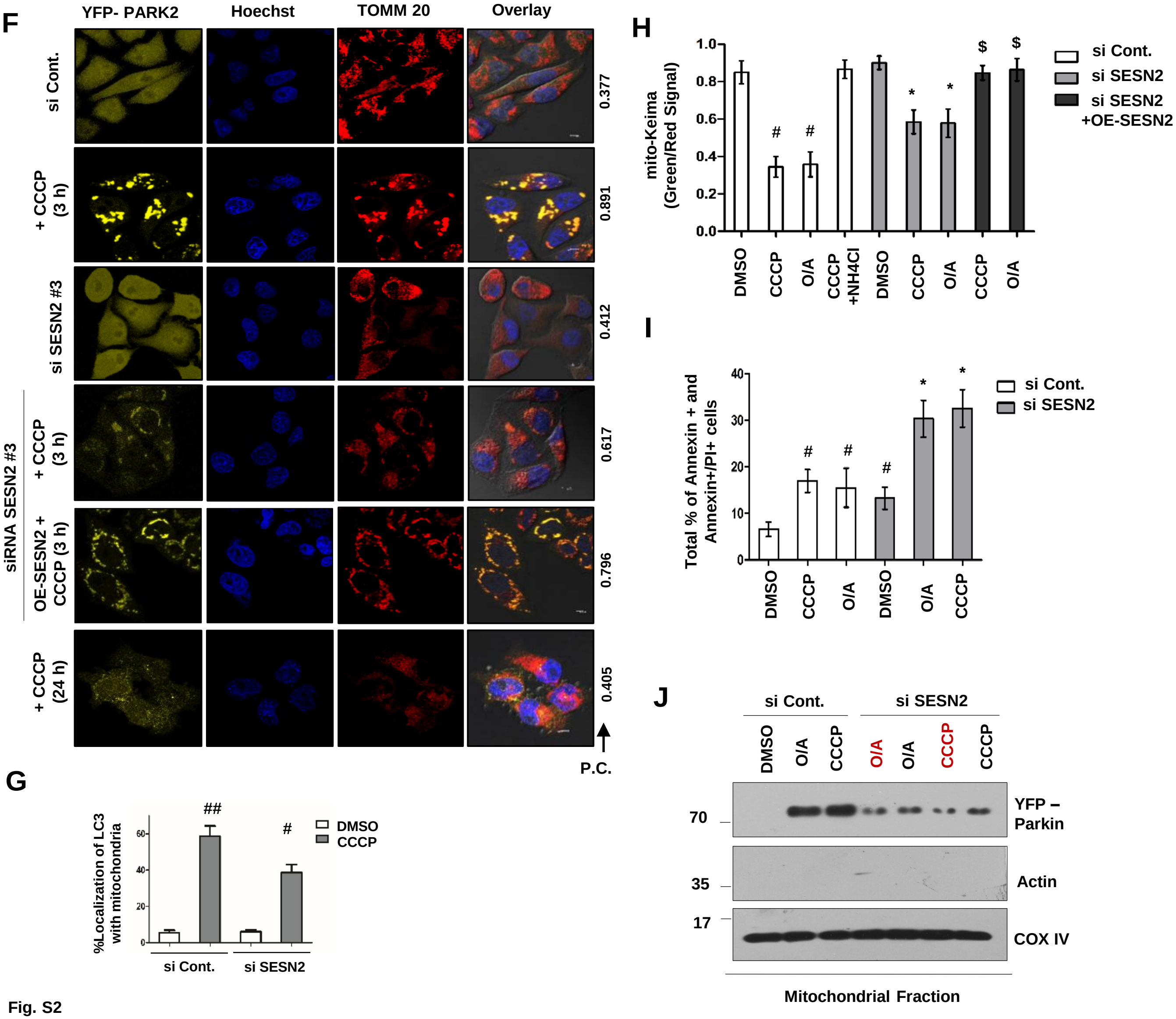

Fig. S2

H. HeLa cells stably expressing with mito-Keima were transfected with myc-Parkin and treatment with 10µM CCCP and 5µM O/A was given for 12 h. Green (458 nm, excitation) and red (560, excitation) signal was acquired and represented as green/red signal. # $p < 0.01$; compared to DMSO (si Cont.), * $p < 0.05$; compared to DMSO (si SESN2), $ $P < 0.05$; compared to CCCP or O/A treatment (si SESN2), NS, non-significant; compared to DMSO (si Cont.)

I. Hela cells were treated with 10 µM CCCP and 5 µM Oligomycin + 5 µM Antimycin A for 2 h in wild-type and SESN2 knockdown cells and total Annexin +ve population (Annexin +ve and Annexin +ve / +Pi+ was measured by flow cytometry. # $p < 0.05$, compared to DMSO (si Cont.), * $p < 0.01$, compared to DMSO (si SESN2.)

J. Wild type and SESN2 knockdown Hela cells stably expressing YFP-Parkin were treated with 10 µM CCCP and 5 µM Oligomycin + 5 µM Antimycin A for 2 h and SESN2 knockdown cells were sorted for Annexin +ve signal (PE- conjugated). Mitochondrial fractions were isolated and translocation of YFP-Parkin was measured. Red, Annexin +ve , Black , Annexin –ve in SESN2 knockdown panel.

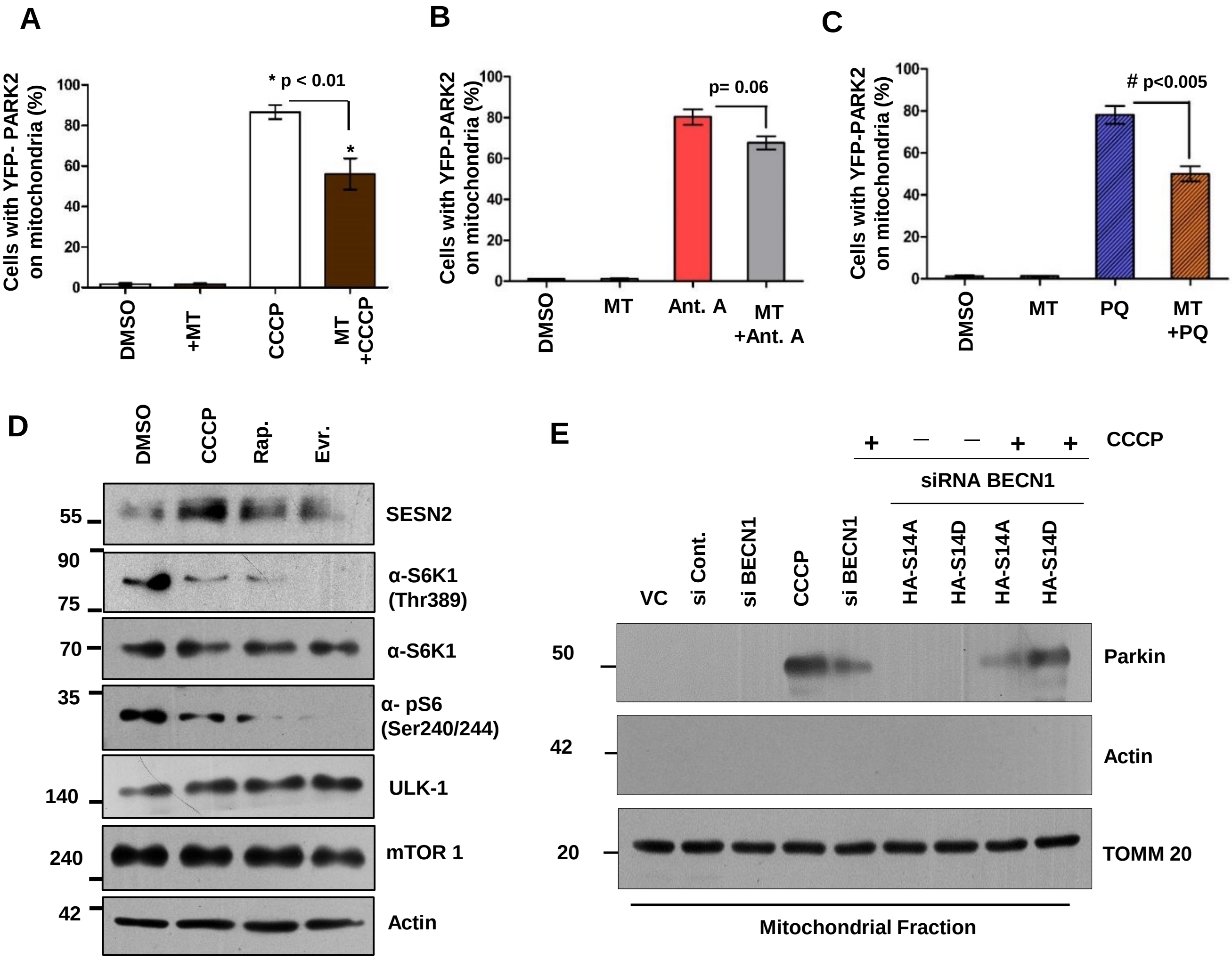

Fig S3

A. HeLa cells stably expressing YFP-Parkin were treated with 10 µM CCCP (2h) and mito-Tempo, percentage of YFP-Parkin co-localizing with mitochondrial marker TOMM20 was assessed. The graph represents mean ± SEM of counts in >100 cells per sample in three independent experiments.

B. HeLa cells stably expressing YFP-Parkin were treated with 40 µM Antimycin A (2 h) and mito-Tempo, percentage of YFP-Parkin co-localizing with mitochondrial marker TOMM20 was assessed. The graph represents mean ± SEM of counts in >100 cells per sample (n=3).

C. Similar to fig. S3A, HeLa cells stably expressing YFP-Parkin were incubated with 5mM Paraquat (2 h) and mito-Tempo, co-localization of YFP-Parkin and TOMM20 was scored and represented in graph as mean ± SEM of counts in >100 cells per sample (n =3).

D. HEK293T cells were treated with 10 µM CCCP, 500 nM Rapamycin and 500 nM Everolimus for 3 h. Analysis of MTOR inhibition and SESN2 levels was done using immunoblotting.

E. Results obtained in fig. 5D were confirmed by analysing isolated mitochondrial fraction from HEK293T cells by immunoblotting using antibodies as indicated. TOMM20, mitochondrial fraction and Actin, cytosolic marker.

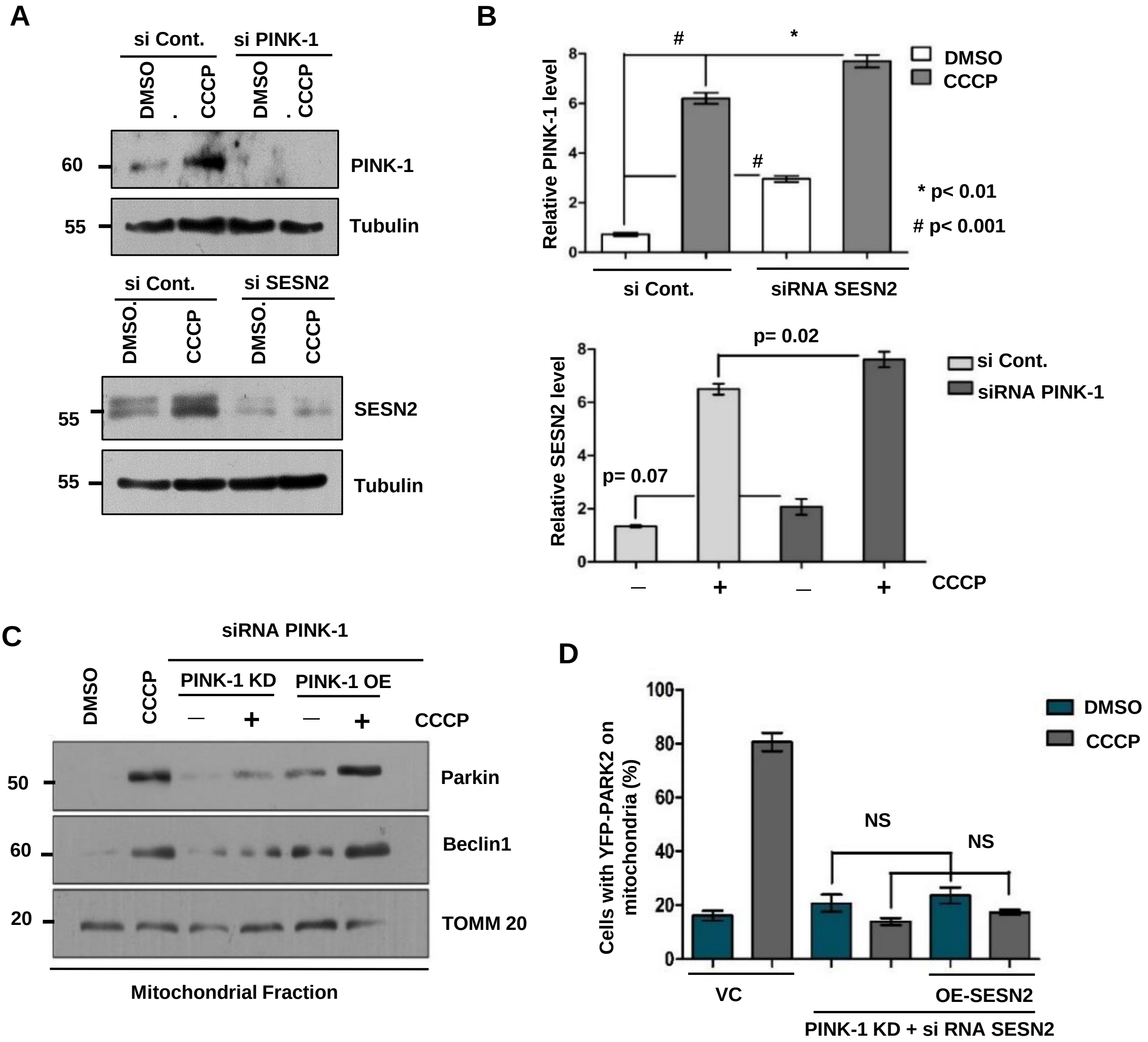

Fig S4

A. Knockdown of SESN2 and PINK1 was performed in HEK293T cells and analysed by immunoblotting using antibodies as indicated.

B. Graphical representation of intensity of SESN2 and PINK1 protein bands, obtained in Fig. 6C. Error bars represent as mean ± SEM (n=3).

C. Cells were transfected with PINK1 siRNA, plasmids expressing PINK1 kinase dead domain and wild type PINK1. Post-transfection cells were treated with DMSO (vehicle) and CCCP for 3 h and mitochondrial fractions were isolated for immunoblot analysis.

D. YFP-Parkin co-localization with TOMM20 in HeLa cells stably expressing YFP-Parkin was scored after counting >100 cells per condition in three independent experiments. The error bars in the graph represent mean ± SEM.

Figure 2 A

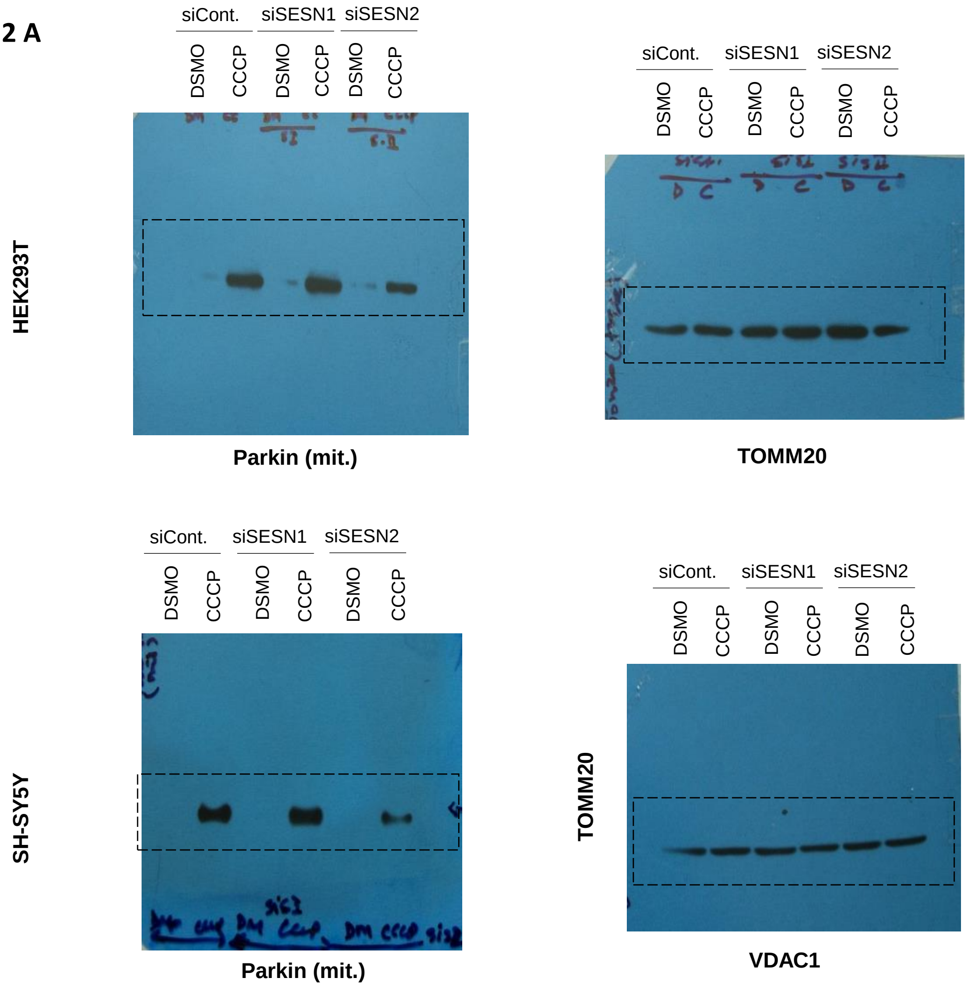

Figure 3C

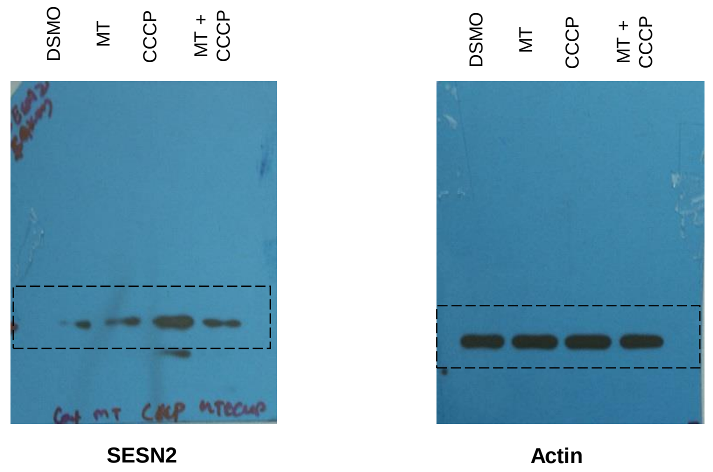

Figure 4C

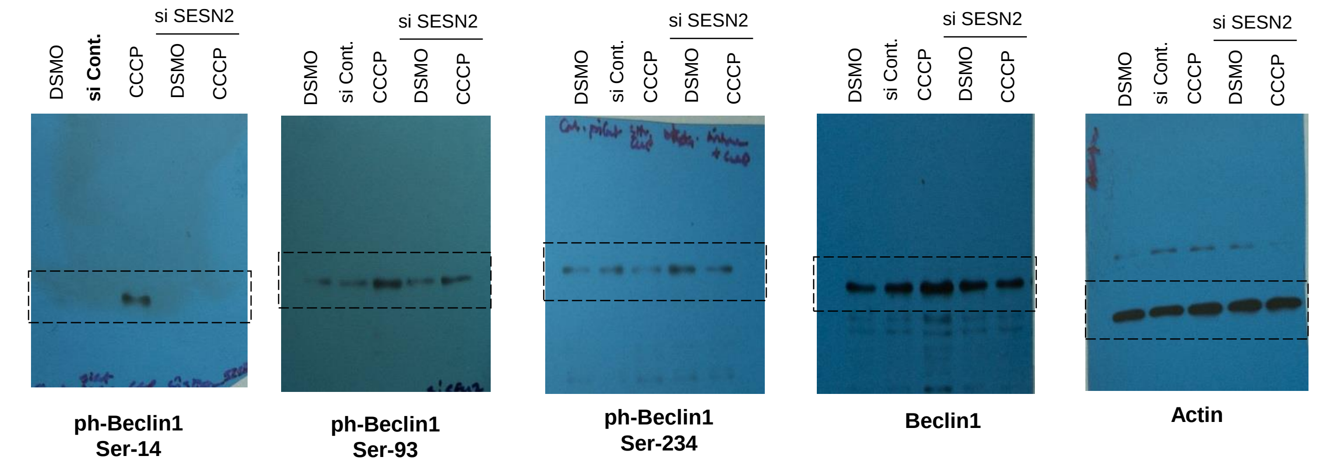

Figure 4D

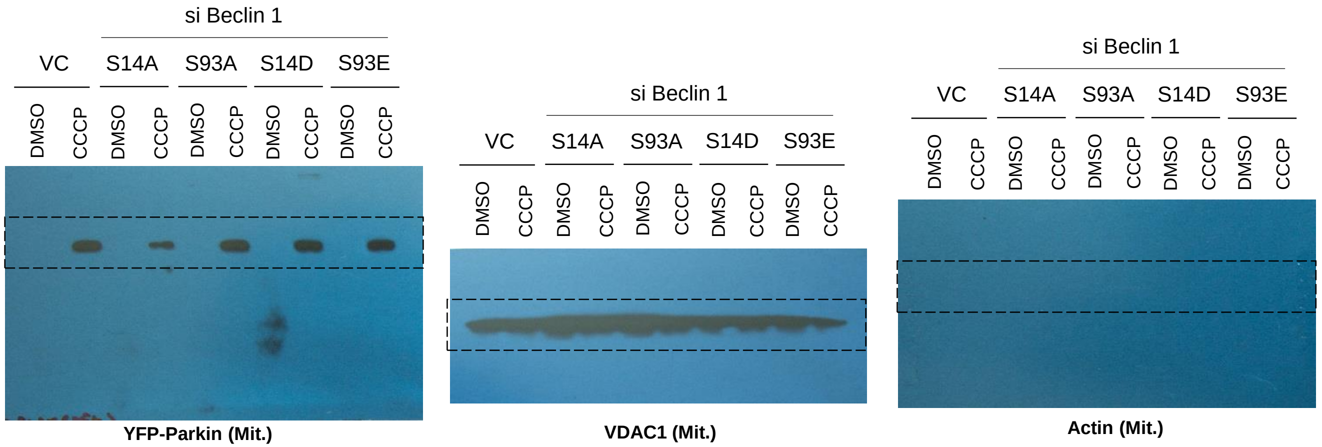

Figure 4E

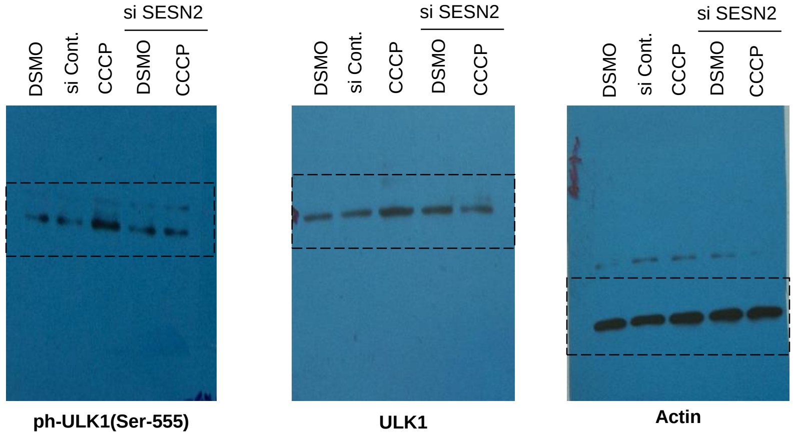

Figure 5A

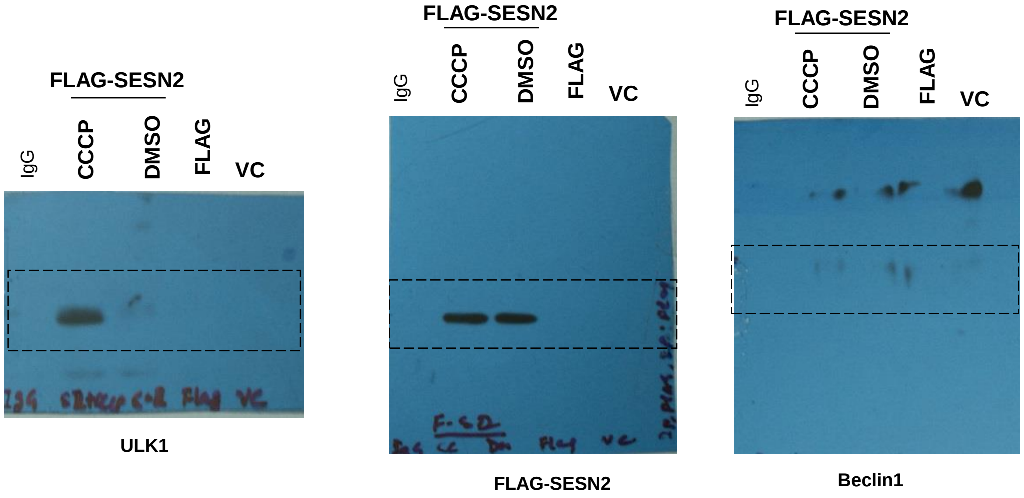

Figure 5E

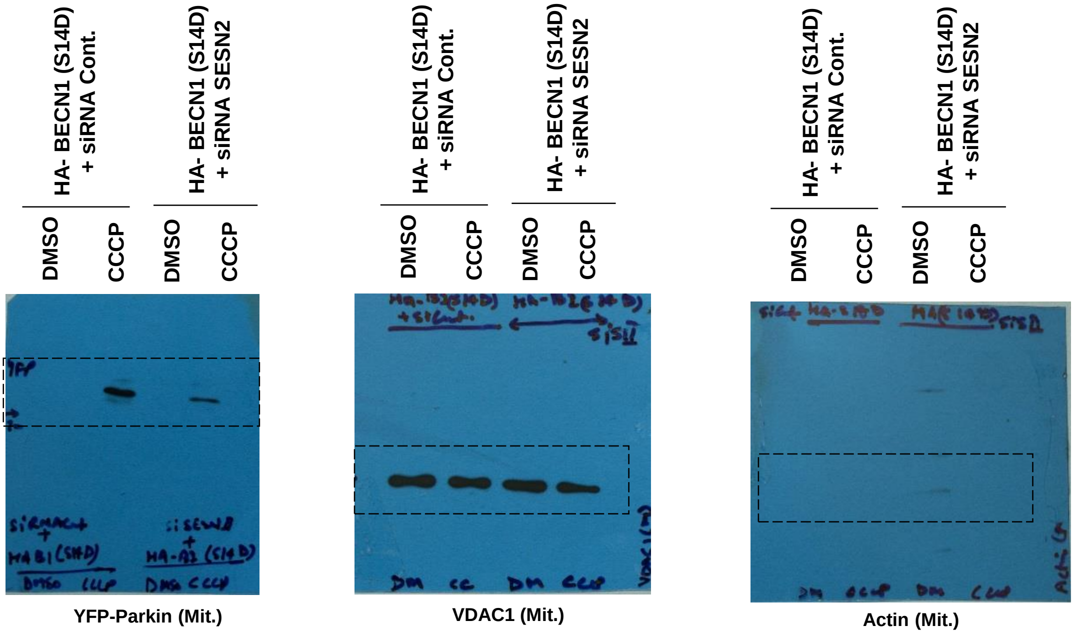

Figure 6D

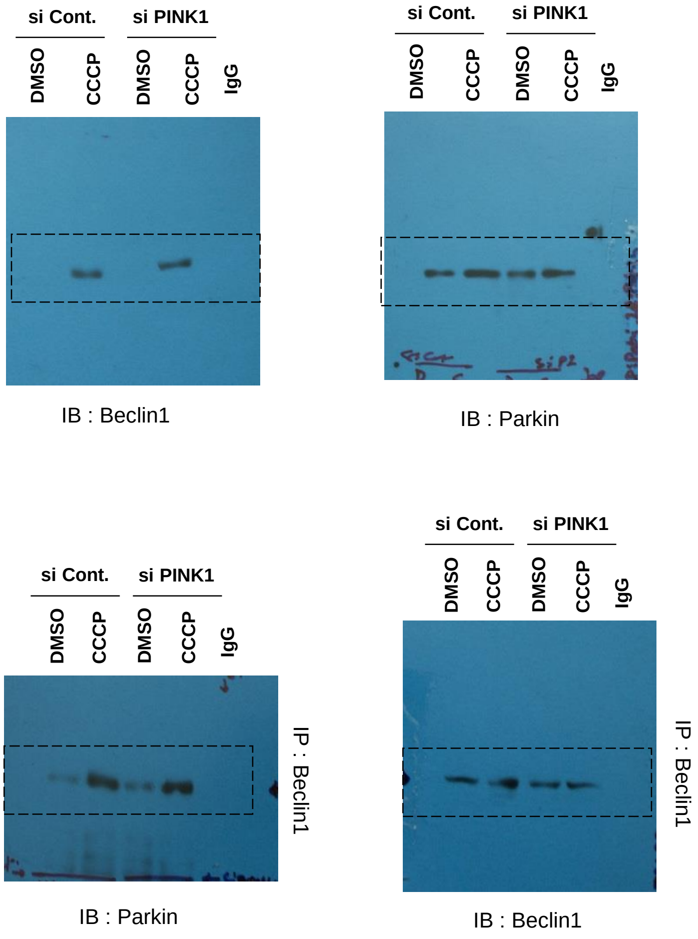
si Cont.
si PINK1
DMSO
CCCP
DMSO
CCCP
IgG
IB : Beclin1
si Cont.
si PINK1
DMSO
CCCP
DMSO
CCCP
IgG
IB : Parkin
si Cont.
si PINK1
DMSO
CCCP
DMSO
CCCP
IgG
IP : Beclin1
IB : Parkin
si Cont.
si PINK1
DMSO
CCCP
DMSO
CCCP
IgG
IP : Beclin1
IB : Beclin1
